# Substantial variation in larval honey bee nutrition within and among *Apis mellifera* colonies

**DOI:** 10.1371/journal.pone.0328027

**Published:** 2026-02-25

**Authors:** Rebecca R. Westwick, Clare C. Rittschof

**Affiliations:** 1 Department of Entomology, University of Kentucky, Lexington, Kentucky, United States of America; 2 Department of Biology, Indiana University, Bloomington, Indiana, United States of America; University of Alberta, CANADA

## Abstract

Parents have evolved strategies to reduce the risk of malnutrition in offspring, including the production of specialized nutritional secretions that are tailored to meet the unique needs of developing offspring. Studies in vertebrates, however, show abundant individual variation in nutritional secretions; the causes and consequences of this variation, and the extent to which such patterns can be generalized beyond vertebrates, remain unclear. Here, we investigated natural variation in nutritional secretions in an invertebrate species, the Western honey bee (*Apis mellifera* L.). This is a unique bee wherein developing larvae subsist entirely on “jellies” (e.g., royal jelly) produced by glands in adult worker bees. We assess within- and among-colony variation in the macronutrient content of the secretions fed to female worker larvae (“worker jelly”), collected from individual larval honeycomb cells. Although female workers make up the largest demographic inside a honey bee colony, very few studies have investigated their larval diet; even fewer have included the scope of colonies needed to assess natural variation in this critical nutritional substance. In one of the largest such studies to date, we found significant variation both within and among colonies in total quantity and macronutrient content of worker jelly, but with greater variation among colonies; this pattern was strongest for proteins. We further assessed whether worker jelly composition was correlated with colony defensive aggression because of extensive links between aggression, foraging activity, and larval development outcomes; however, we observed no such relationship. This study is a critical step in understanding the evolution and maintenance of offspring provisioning strategies, as well as bee foraging ecology and nutritional stress response.

## Introduction

Early-life nutrition has long-lasting consequences for an organism’s health, behavior, and survival, as it is not always possible to later overcome nutritional deficits that occur during this high-growth time [1–3]. Many organisms have evolved parental care to hedge against early-life risks; this often involves adults provisioning young to ensure adequate nutritional intake [4]. Sometimes adults simply assist their offspring in finding naturally available nutritional resources [5]. In other cases, caregivers gather and process nutritional resources before providing them to the young [6]. In the most evolutionarily derived form of provisioning, adults have specialized body structures to synthesize and secrete nutritional products tailored for their young (“socially transferred materials,” such as the milk produced by mammals; [7,8]). Nutritional secretions occur in a wide variety of taxa, including birds, fish, mammals, amphibians, and insects [9–14].

In cases where young feed exclusively on secretions, organisms face the challenge of synthesizing a product that will meet the changing nutritional needs of their offspring across different growth stages [15]. Accordingly, the contents of nutritional secretions are often dynamic and age-specific [16,17]. Despite evidence that nutritional secretions track species- and development-specific nutritional demands, strong inter-individual variation in secretion composition is common, at least in the limited cases where it has been examined [18–23]. This pattern is somewhat surprising [16], as there are costs to variation in secretions; for example, variation in individual human milk oligosaccharides has been correlated with a range of negative health outcomes in infants [24]. The incidence, causes, and consequences of individual variation in nutritional secretions remain unclear.

Studies of variation in nutritional secretions have been conducted almost exclusively in mammalian systems, where one individual (the mother) is typically responsible for all parent-provided nutritional resources. Some social species, notably social insects including the Western honey bee (*Apis mellifera* L., hereafter “honey bee(s)”), distribute brood care labor (such as depositing the synthesized nutritional product “worker jelly” in larval cells) among many hundreds or even thousands of adult relatives [25–27]. There are several potential consequences of this care model, as there may be adaptive benefits to either uniformity or diversity in larval nutrition. For example, if one offspring is exposed to many caregivers, it may average out variation in nutrition received from individual caregivers, resulting in consistent and optimal nutrition and predicting minimal variation across larval nestmates within a social group. Conversely, care from a variety of good and poor providers could increase variation in nutrition among individuals within a social group. It is important to note that in the honey bee, there are cases where individual variation in adult worker bee cue response and behavior confer colony health and resilience [28–34]; if these adult phenotypes are shaped by larval nutrition, high variation in larval nutrition within a colony does not necessarily reflect or result in poor health. Comparing among colonies, if there is a species-specific optimal nutrition target, we may predict minimal variation among social groups [35,36]. However, colony-level differences in resource availability, acquisition rates, and/or adult physiology and behavior could make it challenging to achieve these optimal nutritional targets, resulting in high variation among social groups. In the honey bee, there is evidence that resource availability can greatly impact colony health and productivity [37–39], but few studies have explored corresponding variation in nutrition during the larval “feeding stage,” which is a critical stage for worker bee nutrition [40]. It thus remains unclear how variation in larval nutrition may contribute to colony-level phenotypes, and *vice versa*.

The current study provides one of the largest assessments of worker jelly macronutrient composition (proteins, lipids, and carbohydrates) within and among honey bee colonies to-date. We designed this study to assess whether variation in larval diet could explain a previous finding, that the larval developmental environment impacts adult aggressive behavior in honey bees [41]. Aggression is a complex individual- and colony-level phenotype that reflects genetic variation, but also individual experience, ecological context, and individual and colony health and productivity [42–50]. Our previous work showed that, even among mixed populations of honey bee colonies derived from European sub-species, siblings reared in high-aggression colonies were more aggressive as adults compared to siblings reared in relatively docile colonies. This system offers an opportunity to determine whether variation in a colony-level phenotype that has been tied to health resilience, and accompanying developmental effects, reflects variation in larval nutrition.

In this study, we measured the total amount and macronutrient content of the secretions (“worker jelly”) provided to over 450 individual worker larvae from 18 colonies that show natural variation in defensive aggression. This study represents a critical step towards determining the relationships between developmental nutrition and adult worker phenotypes, as well as the colony-level implications for such patterns. We hypothesized non-significant natural variation in worker jelly macronutrients within and among colonies—especially proteins and lipids that are most critical to larval development [40]—because of the evidence that nurses tailor their secretions to larval stage, and the idea that multiple caregivers can overcome deficits in any single individual’s secretions or variation in the resources available to the colony [51–53]. We additionally hypothesized that high- and low-aggression colonies would show differences in the nutritional makeup of the worker jelly (either in terms of total food available or in terms of macronutrient ratios) because of the established impacts of developmental environment on adult aggression and health [41,48,54]. In contrast to our predictions, we found high levels of variation in worker jelly nutrition both among nestmates within a single colony and among colonies. Although there are some differences in patterns within and among colonies as a function of aggression, the link to nutrition is not easily tied to the quantity of macronutrients.

## Methods

### Study system

Honey bee larvae are fed by a specialized group of adult workers called nurses, which consume pollen and honey and synthesize worker jelly using hypopharyngeal and mandibular glands in the head [55]. Worker jelly secretions are passed orally into honeycomb cells containing developing larvae. The larva typically sits in a pool of worker jelly which she ingests over time. Larvae do not defecate in the food (as honey bees, like most bee species, retain their feces until just before pupation) [56], and the authors are not aware of any evidence that honey bee larvae regurgitate and re-consume their food as some insects do [57]. Previous studies have determined the basic components of worker jelly [58,59] and that the macronutrient profile and other nutritional contents change with larval age [58,60], suggesting precise regulation of nutrition. Several hundred to several thousand nurse bees are active at any given time, walking around the brood nest, checking on larvae, and feeding them if necessary [25]. Nurses care for larvae around the clock, with a single larva being visited upwards of 3,000 times in a single day [61].

### Colony choice and sample collection

Sample collections occurred in central Kentucky, USA from June 25-July 21, 2019. Because previous studies associated colony defensive aggression (hereafter aggression) with nurse care behaviors [54] and larval developmental outcomes [41,48], we sampled worker jelly from colonies ranging in the expression of aggression. We assayed 36 colonies using a previously established aggression assay [41,42]. Briefly, we took photographs at the entrance of the colony and counted the number of visible bees. We then placed a strip of filter paper containing 3 µL of 1:10 isopentyl acetate: mineral oil in the center of the entrance. Isopentyl acetate (IPA) is the main active component of the honey bee alarm pheromone [62], and it provokes workers to emerge from the colony entrance; a greater number of emerging bees signifies higher aggression [47]. One minute following the filter paper application, we took a second photograph to quantify the emergence response. We calculated an aggression score as the difference between the number of bees at the entrance after versus before the filter paper application (this value can be negative if bees retreat in response to the IPA [63]). The 9 highest-aggression and 9 lowest-aggression colonies were chosen for worker jelly collections, blocked across three sites (see below; N = 18 total colonies, N = 3 high-aggression and N = 3 low-aggression at each site, and all colonies at a given site were assayed for aggression on the same day to control for site-level factors like weather that could impact behavior over time).

Each colony has a quantitative aggression score, a relative rank compared to the other 17 colonies in the study, and a categorization as high- versus low-aggression; all measures were incorporated in our data analyses (see below). Quantitative aggression scores can vary substantially due to myriad environmental factors, making it difficult to compare these absolute scores for colonies across studies, locations, or over time. However, the rank order of colony aggression is often consistent [41,42]. The range of raw aggression scores for colonies included in this study (high aggression: 22–165; low aggression: −18–9) is similar to the range included in the previous study that established the link between larval developmental environment and aggression (high aggression: 20–244; low aggression: −16–18) [41]. We performed worker jelly collections within 4–16 days following the aggression assays. This short experimental timeframe (26 total days to identify colonies and collect all samples) minimizes temporal variation in colony and climactic conditions that could influence worker jelly content and/or colony aggression score [64–68].

Most colonies used in this study (N = 10) originated from packages in late April 2019 with strains advertised as “Italian” and “Russian Hybrid” (Schoolhouse Bees, Covington, KY). Two colonies were installed as packages in May 2019 from two separate providers (Dadant & Sons, Frankfort, KY; Hosey Honey, Midway, KY) and no strain information was provided. Remaining colonies (N = 6) were locally open-mated and previously overwintered as mature hives. Colonies were kept at one of three sites. All sites were on mixed-use farms with similar surrounding landscapes. Two of the sites were approximately 1.6 km apart (“Alpha” and “Beta”), which is within the maximum honey bee foraging range but beyond the typical range of most foraging trips [69–72]. A third site (“Gamma”) was approximately 14.5 km away from the other two sites, which is outside of the maximum foraging range for Alpha and Beta [73]. Variation in the surrounding landscape could impact the floral resources available to colonies (and thus worker jelly content); though we did not perform any direct assessments of landscape floral resources in this study, we do consider location as an explanatory variable in our analyses.

The macronutrient content of the worker jelly changes with larval age [58]. We therefore sampled from age-matched larvae. Trapping the queen against appropriately sized honeycomb for a short period of time is a common strategy to generate age-matched worker offspring [74,75]. For each of the 18 colonies, we caged the mother queen with empty honeycomb for 24 hours to allow her to lay worker-destined eggs. The cage fit one standard wooden “deep” frame with worker-brood-sized honeycomb from a Langstroth beehive (~48 cm x 3 cm x 23 cm). Two sides of the cage were covered by a “queen excluder,” a plastic grate that was large enough to allow adult workers to pass in and out freely and tend to the young while being small enough to contain the larger queen [76,77]. After 24 h, the queen was released back into the colony, and the frame was placed back in the colony within the cage to prevent further laying by the queen. This approach generates dozens to hundreds of eggs that range in age from 0 to 24 h old.

Nurses begin making feeding visits to deposit worker jelly almost immediately once a larva hatches, which takes about three days following egg laying [61,78]. Two days after the eggs hatched (96–120 hours post-laying), we removed frames to collect worker jelly on a single day per colony between 9:00 and 11:00. Samples from all 18 colonies were collected on one of 8 sampling days (S11 Table). We brushed off adult bees and covered the frame with damp paper towels to prevent larva and worker jelly desiccation. We carried the frame into a building to collect the worker jelly (<5 min following frame removal). We haphazardly chose approximately 27 cells covering the entire laying area on one side of each frame, excluding any cells where the larvae were markedly large or small or had any abnormalities. We collected 467 total samples across the 18 colonies. Some colonies (N = 4) had fewer than 27 samples due to an insufficient number of usable larval cells on one side of the frame; this was usually the result of poor laying success by the queen.

Honey bee larvae sit in the base of honeycomb cells in a small pool of worker jelly. To collect the worker jelly, we pipetted 100 uL of deionized water into the cell which caused the larva to float to the top. We carefully removed the larva with a small metal queen grafting tool and placed it in a 1.5 mL microcentrifuge tube (Thomas Scientific), which we stored at −80⁰C. We then pipetted an additional 100 uL of deionized water into the cell (200 uL total). We drew the slurry of water and worker jelly in and out of the pipette several times to mix it and to loosen the jelly from the sides of the cell in order to collect as much of the jelly as possible. We pipetted the mixture into another 1.5 mL microcentrifuge tube (Thomas Scientific), where it was stored at −80⁰C until chemical processing.

### Sample processing

#### Total wet and dry mass.

Worker jelly has a gelatinous texture and is comprised of water, proteins, lipids, carbohydrates, and other material such as micronutrients and nondigestible material like fiber. To homogenize the worker jelly samples and break up clumps, we sonicated the samples for 5 min on 100% power with a Misonix S-4000 cup horn sonicator (Misonix, Newton, CT). We then determined the wet and dry mass of a 20 uL aliquot from each worker jelly sample and used these values to calculate total wet and dry mass for each sample. Samples were air dried in a low-humidity room for 24 h and weighed on an analytical balance (XSE105 Dual Range with 0.01 mg resolution, Mettler Toledo, Columbus, OH, USA).

#### Protein quantitation.

We used a bicinchoninic acid (BCA) assay to quantify total protein mass per sample (Pierce™ BCA Protein Assay Kit, 23227, Thermo Fisher Scientific, Waltham, MA, USA). Note that the BCA assay, while robust to variation in amino acid composition, can be affected by polyphenol presence in samples [79], which is common in bee-collected plant products like nectars, pollens, and jellies [80–82]. While this assay has been routinely used to measure protein content in bee pollens and jellies [83–86], results and especially comparisons with other studies should be interpreted with this caveat in mind. The assay was carried out according to the manufacturer’s instructions for a microplate preparation, with Bovine serum albumin (2 µg/µL) as the standard. The standard curve included volumes of 0 µL, 2.5 µL, 5 µL, 7.5 µL, 10 µL, and 20 µL of the standard. We measured protein from a sample fraction unique from the fraction used for the lipid and carbohydrate extractions (see below). We adjusted the volume of the starting fraction for each sample based on the sample’s total dry mass to ensure the protein readings fell within the assay’s standard curve; we accounted for this variation in calculations of total protein mass per sample (mg). We measured all samples in triplicate. Samples were randomly distributed across assay plates to avoid any technical bias caused by plate effects, though an individual sample’s triplicates were all on the same plate. The final color is stable for approximately half an hour; it was during this half-hour window that we measured the absorbance on a microplate reader at 562 nm according to the manufacturer’s instructions (CLARIOstar Plus, BMG LABTECH, Offenburg, Germany). We used the median triplicate value of each sample to calculate the total protein mass per sample (mg). We also divided total protein mass by total dry mass to calculate the relative protein quantity per sample (a unitless value).

#### Lipid and carbohydrate quantitation.

Lipid and carbohydrate levels were measured from a single 100 µL aliquot on which we performed combined chloroform-methanol extraction and fractioning (following [87]). We performed a sulfo-phospho-vanillin assay on the chloroform fraction to determine the amount of lipids per sample [87,88]. This assay detects unsaturated fatty acids, which comprise the majority of lipids in honey bee jellies (primarily 10-HDA [89]), but is notably insensitive to the minor saturated fatty acid and sterol content [90]. We hereafter refer to our lipid results as “total lipids” to concisely differentiate it from our measure of “relative lipids” (normalized to the total dry weight of the sample), but both the “total” and “relative” values are more precisely described as total/relative unsaturated fatty acids. Commercial vegetable oil (primarily soybean, with an unsaturated fatty acid content of ~85–88% [91]) suspended in chloroform (1 µg/µL) was used as the standard, with volumes of 0 µL, 12.5 µL, 25 µL, 50 µL, and 100 µL for the standard curve.

An anthrone-sulfuric acid assay was used on the methanol fraction to determine carbohydrate concentrations [87,92]. Anhydrous glucose dissolved in deionized water (1 µg/µL) was used as the standard for this assay, with the same range of volumes for the standard curve. As with the protein assays, we adjusted the volume of the starting fraction for each sample based on the sample’s total dry mass to ensure the readings fell within the assay’s standard curve; we accounted for this variation in calculations of total lipid and carbohydrate mass per sample (mg).

Samples were once again run in a random order. We also measured these samples in triplicate, although due to the nature of the assay, the samples were not split into three replicates until after the color change had occurred (technical replicates rather than biological replicates—biological replicates were precluded by low total sample volumes). Like the protein assays, we measured the absorbance on a CLARIOstar microplate reader during the half-hour window that the color change is stable. The median value of each triplicate was used to calculate total lipid mass per sample (mg) and total carbohydrate mass per sample (mg), as well as relative lipid and carbohydrate quantities (unitless) as described above for protein.

### Statistical analyses

We performed statistical analyses with R version 4.1.2 [93]. Our analyses primarily focused on total dry mass (mg); total protein, lipid, and carbohydrate mass (mg); and relative protein, lipid, and carbohydrate quantities (unitless) as response variables. To run a Principal Components Analysis (PCA) on macronutrient masses and relative quantities, we used the prcomp() function in the “stats” package [93]. The “ggbiplot” package was used to create visualizations of the PCA [94].

We used the “lme4” package in R to create separate linear mixed models (LMMs) to assess the fixed effects aggression, colony site, and/or colony genetic strain separately for each response variable (macronutrient masses and relative quantities) [95]. We incorporated aggression into the analyses in three ways (with separate models for each): as a binary variable (high/low), as a ranked variable (1–18), and as a continuous variable using quantitative aggression scores. Colony site and colony genetic strain each had three levels (Alpha, Beta, or Gamma; and “Italian,” “Russian hybrid,” or open-mated). Square-root and log + 1 transformations were used as needed to normalize the data and improve model fit. Total dry mass was either square root or log + 1 transformed depending on the analysis (stated individually in the RESULTS). Unless otherwise stated, protein, lipid, and carbohydrate total masses and relative quantities were square-root transformed. Our initial models each contained a single fixed effect (aggression level, aggression rank, quantitative aggression score, colony site, or genetic strain) and colony identity as a random effect. We additionally created one model that included multiple fixed effects: aggression, colony genetic strain, and their interaction, with colony ID as a random effect. We used the DHARMa package to assess the quality of model fit, which included a Q-Q plot, Kolmogorov–Smirnov test, dispersion test, outlier test, group-level uniformity test, and Levene test [96]. We used Type II ANOVAs to assess significance values for our models; for this we used the “car” package [97]. To calculate among-colony variance, we used the “aov” function from the “stats” package to run ANOVAs [93]. To perform median-centered Levene’s tests for homogeneity of variance, we used the “leveneTest” function from the “car” package [97]. Figures were created using the “ggplot2,” “ggpubr,” “cowplot,” and “viridis” packages [98–101].

### Ethics statement

No approvals, licenses, or permissions were required to carry out this work. All honey bee colonies used in this study were maintained according to best practices recommended by the Honey Bee Health Coalition.

## Results

### General observations and relative macronutrient patterns among samples

We measured macronutrients in 467 samples of worker jelly from 18 different colonies. Our samples had an average total dry mass of 1.24 ± 0.63 mg (mean ± SD) with 0.82 ± 0.50 mg of total proteins (but see METHODS), 0.05 ± 0.03 mg of total lipids (unsaturated fatty acids, see METHODS), 0.10 ± 0.06 mg of total carbohydrates, and 0.26 ± 0.34 mg of other matter (e.g., nondigestible material, micronutrients, minerals, contaminants, etc.). These measurements give an average ratio of approximately 16:1:2:5 proteins:lipids:carbohydrates:other matter, or 66.6% proteins, 4.4% lipids, 7.9% carbohydrates, and 21.1% other matter. Notably, the ratio of proteins to lipids could be an overestimate due to measurement limitations (see METHODS). S12 Table shows how these values compare with previous studies that measured worker jelly composition. There is considerable variation in relative nutrient composition among studies, likely due to a combination of methodological differences, differences in the ages of the larvae measured, historical changes in diet and nutritional resources (studies cover a 100-year span), and geographic location (multiple continents are represented among studies).

**Fig 1:**
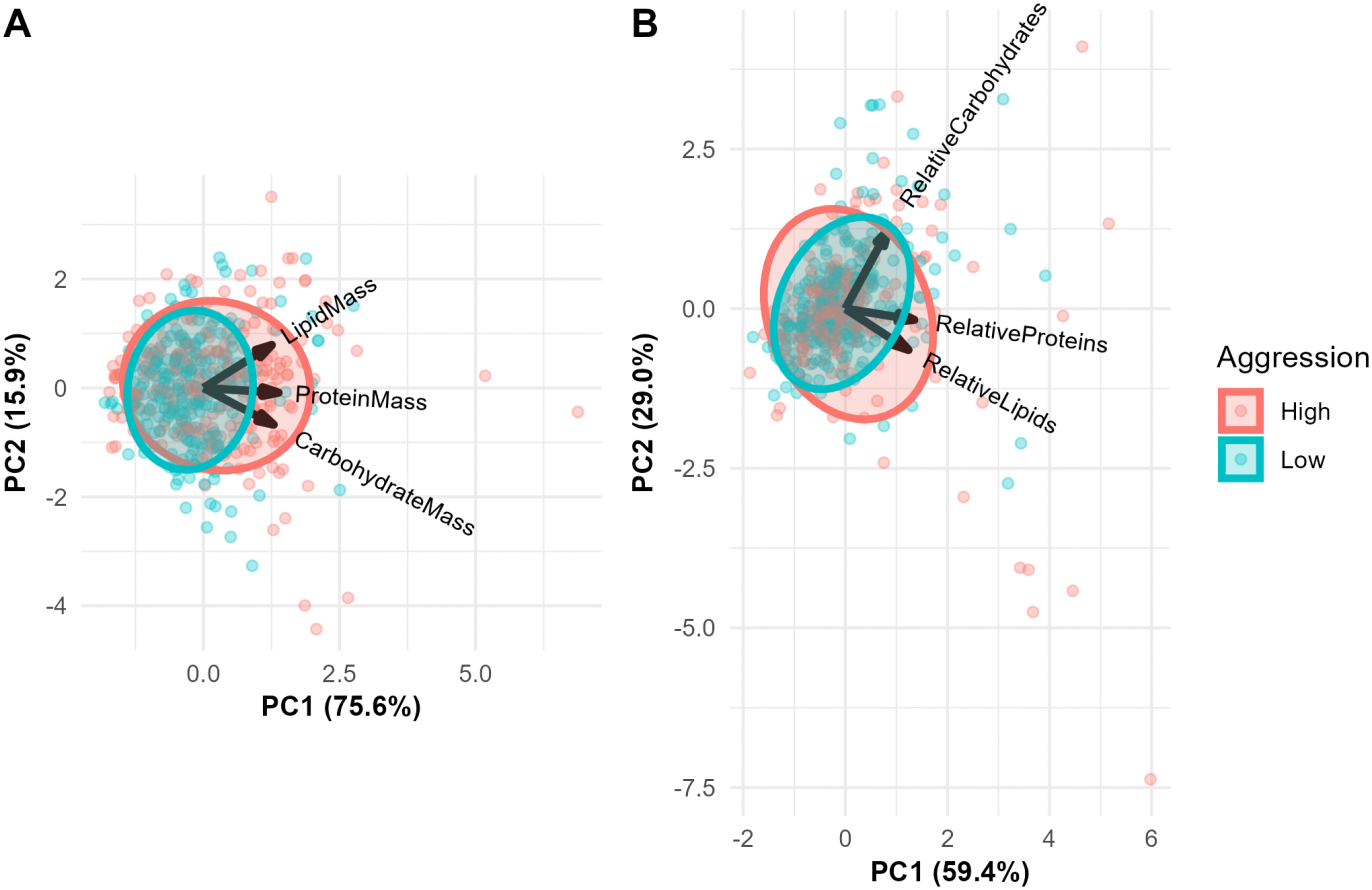
Principal Component Analysis of (A) total protein, lipid, and carbohydrate mass (mg) as well as B) relative quantities with ellipses showing colony aggression level (high in red vs. low in blue). PC1 for both total and relative quantities is largely driven by protein, while PC2 loads lipids and carbohydrates opposite of one another. Note high degree of overlap between aggression levels, with slightly more variation in high-aggression colonies along PC1 of **A)**.

We assessed the diet constituents driving relationships among samples using a Principal Component Analysis (PCA). We performed two analyses, one using macronutrient masses and one using relative macronutrient quantities (omitting the “other” category; Fig 1). For mass, the first component (PC1), which was largely attributable to variation in protein, explained 75.6% of the total variance. The second component (PC2) explained 15.9% of the variance for a total of 91.5%. PC2 depicted an inverse relationship between lipids and carbohydrates. The PCA for relative macronutrient quantities gave similar results, but with slightly less total variance explained (88.5%), and greater evidence of covariation between lipids and proteins.

### Variation within and among colonies in worker jelly quantity and content

**Fig 2:**
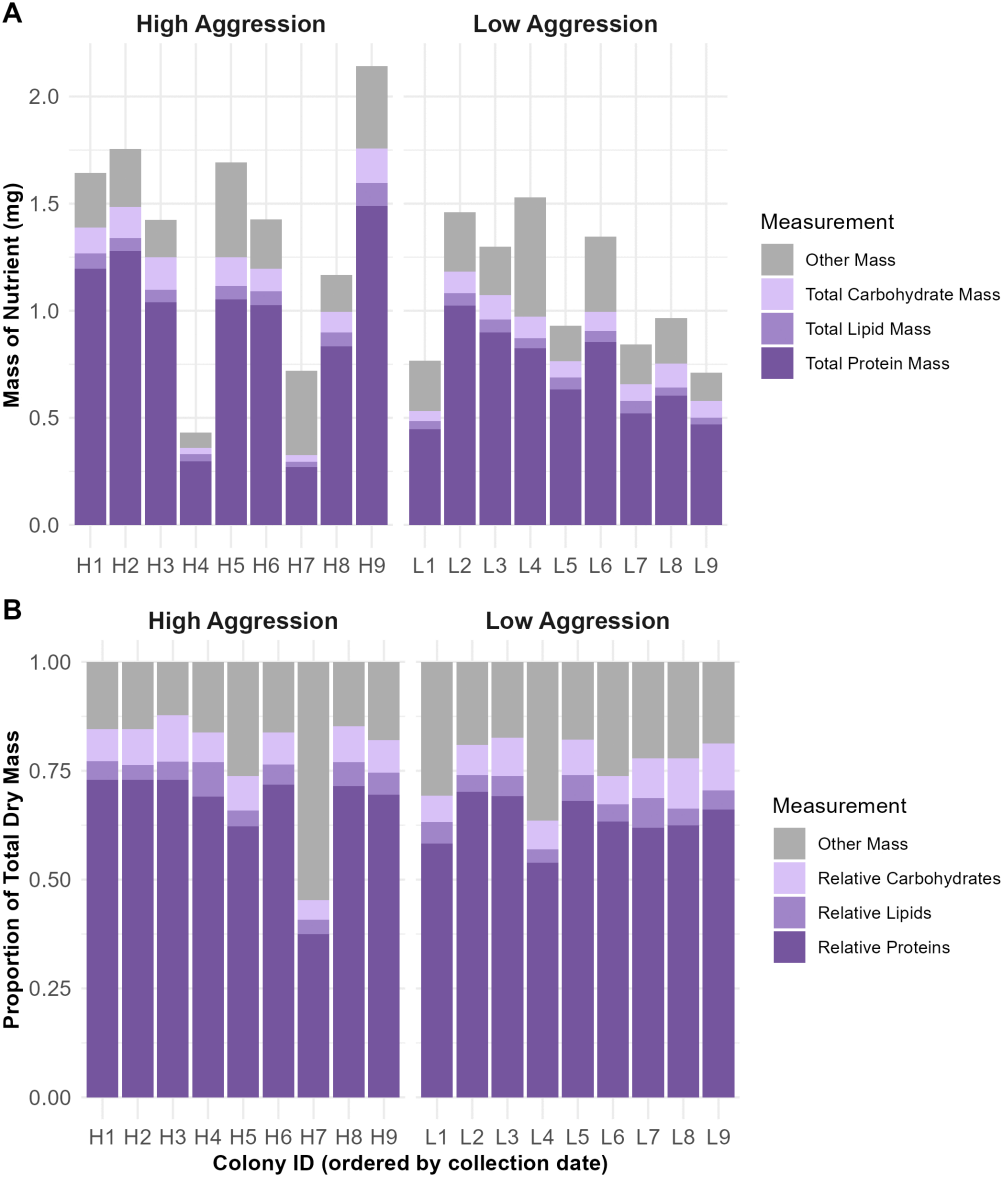
Colony variation in total dry mass, and total and relative mass of each macronutrient. High-aggression colonies (H) are clustered on the left and low-aggression colonies (L) are clustered on the right in each panel. A) Per-colony mean total mass per sample of each macronutrient (mg). The total height of each bar is the mean total dry mass per sample (mg). B) Per-colony mean proportion of each macronutrient relative to the total dry mass of each sample (which is set at 1).

There was considerable variation among colonies in the mean total dry mass per larval sample; colony means ranged from 0.4–2.1 mg (Fig 2A). Mean total mass of each macronutrient also varied substantially among colonies (proteins: 0.27–1.49 mg, lipids: 0.02–0.11 mg, carbohydrates: 0.03–0.16 mg), as did relative macronutrient quantities to a lesser degree (Fig 2B). Variance among colonies was higher than within-colony variance among larvae for total dry mass and total mass of each macronutrient (ANOVA: total dry mass (square-root transformed): F_17,449_ = 24.4, P < 0.0001; total protein mass: F_17,449_ = 26.1, P < 0.0001; total lipid mass: F_17,449_ = 17.3, P < 0.0001; total carbohydrate mass: F_17,449_ = 20.1, P < 0.0001). All three macronutrients varied significantly among colonies, which means that diets among larvae within a colony are similar enough to detect colony-level differences. Total protein mass showed the biggest difference among colonies relative to its level of within-colony variation, followed by carbohydrates and lipids (indicated by the size of the F-statistic).

Within-colony variance among nestmates was also substantial in some cases. Colony coefficients of variation across larvae for each macronutrient (expressed as total macronutrient mass) ranged from 0.26–0.82 (S1 Fig). S2 Fig displays the ranges of macronutrient masses among nestmates for each colony in this study. At the most extreme, there was a colony with a 10-fold range in proteins (Colony H1: 0.43 mg-4.14 mg), a colony with a 14-fold range in lipids (Colony L5: 0.008 mg-0.11 mg); and a colony with a 22-fold range in carbohydrates (Colony L2: 0.01 mg-0.23 mg). Relative macronutrient quantities showed similar range magnitudes (proteins: Colony L1, 16-fold; lipids: Colony H4, 28-fold; carbohydrates: Colony L7, 11-fold). These results indicate that—while within-colony variance is lower than among-colony variance—the larvae at the extreme ends within a given colony may still be receiving substantially different nutrition from one another.

Patterns of variation within colonies were most dynamic for proteins. Total protein mass showed significant non-homogeneity of variance among colonies, meaning that some colonies showed significantly higher within-colony variance than others (Levene’s test: total protein mass: F_17_ = 2.76, P = 0.0002). No other macronutrient showed this pattern (Levene’s test: total lipid mass: F_17_ = 1.36, P = 0.15; total carbohydrate mass: F_17_ = 0.91, p = 0.56), and neither did total dry mass (Levene’s test: total dry mass square-root transformed: F_17_ = 0.88, P = 0.60). Overall, protein appears to be the macronutrient that has the greatest variation, both in terms of differences among colonies, and the patterns of variation among larvae within colonies.

### Relationship between colony aggression and worker jelly quantity, content, and variance

**Fig 3:**
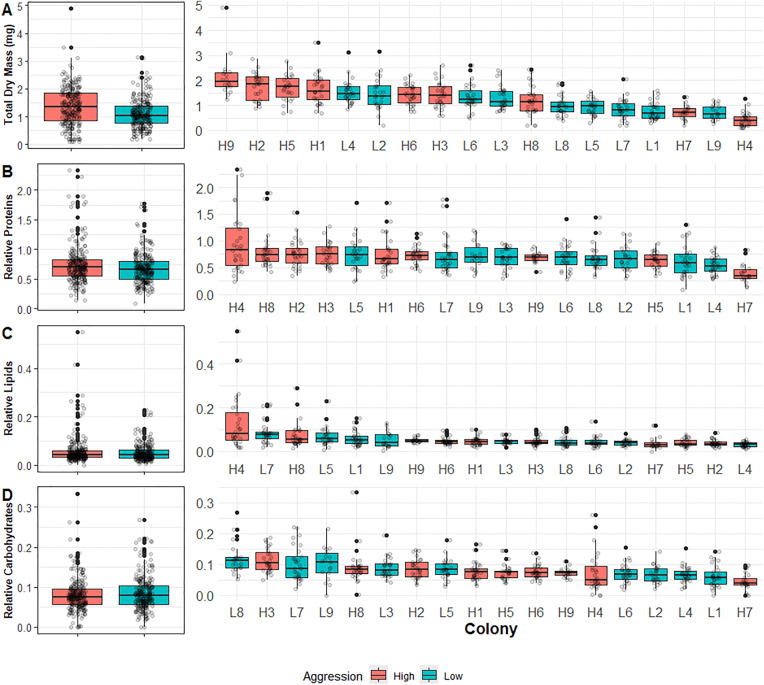
Total dry mass and relative amounts of each macronutrient in worker jelly as a function of colony aggression level (High versus Low) and individual colony. Boxplots show the distribution (1^st^ quartile, median, and 3^rd^ quartile for the box and 1.5x the IQR for the whiskers). The left-side panels show all colonies pooled by aggression level, while the right-side panels show the same data separated by individual colony for A) total dry mass (mg), B) relative proteins, C) relative lipids, and D) relative carbohydrates. Each dot represents a unique worker jelly sample. S6 Fig additionally shows panels for total proteins, total lipids, and total carbohydrates (all mg) instead of the relative amounts displayed here.

Aggression scores for all 18 colonies are shown in S3 Fig. To assess the relationship between colony aggression and worker jelly quantity and content, we first treated aggression as a binomial variable (low versus high) in LMMs. This analysis showed no association between colony aggression and total dry mass or relative macronutrients quantities (Fig 3; total dry mass (log-transformed): Wald X^2^_1_ = 1.3, P = 0.25; relative protein quantity: Wald X^2^_1_ = 0.64, p = 0.42; relative lipid quantity: Wald X^2^_1_ = 0.07, P = 0.79; relative carbohydrate quantity: Wald X^2^_1_ = 0.42, P = 0.51). Similar models with total macronutrient masses (mg) gave comparable results (S4 Fig), as did analyses that treated aggression as either a ranked variable (1-18) or a continuous variable (see METHODS; S5-S7 Figs; [41]). Fig 1 shows a Principle Component Analysis (PCA) of both total and relative macronutrients as a function of aggression level; there was very little separation between high- and low-aggression colonies in this analysis (Fig 1).

Because the high-aggression colonies included some of the highest and lowest per-colony mean total dry masses (Fig 2), we assessed how within- and among-colony variance in worker jelly differed between high- and low-aggression colonies. Similar to the whole-dataset analysis described above, we used ANOVAs for total dry mass and relative macronutrient quantities within each aggression level to test how within-colony versus among-colony variance changed as a function of aggression level. We identified significant among-colony variation for each macronutrient within both aggression levels (ANOVA: total dry mass (square root-transformed): high: F_8,225_ = 33.6, P < 0.0001; low: F_8,225_ = 13.5, P < 0.0001; relative protein quantity: high: F_8,225_ = 38.1, P < 0.0001; low: F_8,225_ = 10.4, P < 0.0001; relative lipid quantity: high: F_8,225_ = 28.4, P < 0.0001; low: F_8,225_ = 6.5, P < 0.0001; relative carbohydrate quantity: high: F_8,225_ = 33.5, P < 0.0001; low: F_8,225_ = 7.5, P < 0.0001).

The magnitude of the F-statistic, which reflects the ratio of among-colony to within-colony variance, was much higher for the high-aggression colony set for total dry mass as well as relative macronutrient quantities. This indicates that among-colony nutrient variation was more substantial for high- versus low-aggression colonies.

### Alternative hypotheses to explain variation in worker jelly content

#### Does diet change over our experimental timeframe?

We designed our experiment to assess whether colony aggression level explained variation in worker jelly content, but we performed some additional analyses to assess other possible contributing factors. We first assessed evidence of variation over the course of our experimental timeframe. An ANOVA across the 8 sample collection days (see METHODS) showed that collection date did not explain significant variation in total dry mass or macronutrient quantities (S8 Fig; total dry mass (square root-transformed): Wald X^2^_1_ = 0.81, P = 0.37; relative protein quantity: Wald X^2^_1_ = 0.25, P = 0.61; relative lipid quantity: Wald X^2^_1_ = 0.33, P = 0.56; relative carbohydrate quantity: Wald X^2^_1_ = 0.42, P = 0.52).

#### Does diet vary across sites?

Next, we assessed evidence of site-level variation in worker jelly. We found that site was not associated with significant variation in in any of our measures (LMMs with site as a fixed effect and Colony ID as a random effect; total dry mass (log-transformed): Wald X^2^_2_ = 2.9, P = 0.24; relative proteins: Wald X^2^_2_ = 0.7, P = 0.70; relative lipids: Wald X^2^_2_ = 2.6, P = 0.28; relative carbohydrates: Wald X^2^_2_ = 1.4, P = 0.50, S9 Fig). Results were similar for total macronutrient masses (data not shown).

#### Does diet vary as a function of genetic strain?

Finally, we examined effects of colony genetic background on worker jelly content, focusing on 411 samples from 16 colonies representing three genetic “strains”: those advertised as “Italian” (N = 4) or “Russian Hybrid” (N = 6), and “Open-mated” colonies from a local beekeeper (N = 6) [102]. Two additional strains (each with N = 1 colonies) were excluded due to low replication (see METHODS). We found no evidence of significant variation in worker jelly total dry mass or relative macronutrient quantities as a function of genetic strain (LMM, total dry mass (log-transformed): Wald X^2^_2_ = 5.6, P = 0.06; proteins: Wald X^2^_2_ = 0.9, P = 0.64; lipids: Wald X^2^_2_ = 2.5, P = 0.29; carbohydrates: Wald X^2^_2_ = 2.5, P = 0.29, S10 Fig). Results were similar for total macronutrient masses (data not shown). Because aggression is partially heritable [103–105], we assessed evidence for an interaction between aggression level and strain. We did not detect statistically impactful collinearity between aggression and genetic strain (generalized variance inflation factor (GVIF)—all values below 2). We therefore ran an additional model that included both genetic strain and aggression as well as an interaction term between aggression and genetic strain, but this model yielded similar nonsignificant results (data not shown).

## Discussion

In one of the largest modern studies of honey bee worker jelly content (over 450 samples from 18 unique colonies), we observed substantial variation for age-matched samples; variation among colonies was significant, suggesting comparatively modest variation among larvae within colonies. We assessed whether colony aggression level predicted variation in larval nutrition and found that high-aggression colonies show more extreme among-colony variation than low-aggression colonies. However, aggression level was not a straightforward predictor of total diet quantity or any single macronutrient. Further analyses of temporal, site, or genetic strain effects did not reveal any significant predictors of variation in larval diet among colonies.

Proteins, lipids (unsaturated fatty acids), and carbohydrates all showed significant among-colony variation in our study. Protein makes up the greatest proportion of worker jelly, and it is often discussed as one of the most critical components of insect larval development [106–109]; proteins not only showed the greatest among-colony variation, but colonies also differed substantially in the extent to which samples from different nestmates varied in this nutrient. It is important to consider that the BCA analysis we used to measure total protein may respond to the presence of polyphenols in the diet, which may introduce unaccounted variation to our measurements. Lipids, the smallest worker jelly component, showed the smallest differences among colonies relative to differences within colonies (although still significant colony-level variation). Carbohydrates, arguably the most easily acquired macronutrient due to its presence in both pollen and nectar/honey, showed an intermediate level of among- relative to within-colony variation [40,110].

Surprisingly, even with multiple caregivers to potentially “average out” the nutritional input to larvae, the worker jelly nutritional secretions fed to age-matched larvae within a single colony showed high variation, even higher than what is seen in species that are provided for primarily by a single maternal caregiver, e.g., mammals. For example, in studies of human and goat milk, the fold changes between the highest and lowest observation for a given macronutrient were typically in the range of 1–4. We observed fold-change differences of 11–28 (relative quantities) *within single colonies* at the most extreme end (although the total number of observed samples was somewhat lower for both mammalian milk studies, possibly hiding some variation) [18–20]. Despite this relatively high within-colony variance, among-colony variance was higher, indicating that a complex combination of factors, e.g., floral resource availability, the size and health of the nursing cohort, and/or genetic and environmental variation in provisioning strategy likely impacts the nutritional experience of individual worker honey bee larvae.

Variation in lipid and protein content is surprising given their critical role in larval development, and the evidence that these nutrients serve as dietary choice criteria in other bee species ([111–113], but see also [114]). One possibility is that, once a minimum macronutrient threshold is met, any further variation is less important for health and fitness consequences; quantities exceeding a minimum level would not necessarily be selected against [115]. Honey bee larvae could also represent what is referred to in nutritional ecology as “macronutrient generalist”—that is, a species that is able to physiologically tolerate a wide range of macronutrient ratios [116,117]. Overall, though recent research has suggested that nurse bees may regulate their dietary intake of protein-to-lipid ratios [113], our work suggests that this regulation does not necessarily lead to a species-specific, stable protein-to-lipid ratio in the worker jelly. However, few other studies have extensively sampled macronutrients from worker jelly, nor determined the outcome of nutritional variation; data on lipids are particularly scarce (S13 Table). Many more studies are thus required to understand the nutritional needs of honey bee larvae, including minimum macronutrient requirements and the ways in which nurse bees may respond to and accommodate resource shortages and variation.

We found that the ratio of proteins to lipids in worker jelly is about 16:1. Although our estimate is likely an overestimate due to our measurement approaches (METHODS), data from other studies on honey bee larval jellies also suggest a substantially higher protein:lipid ratio compared to the ratios identified in honey-bee-collected pollens (1.5:1), even when using similar macronutrient measurement methods [112,118–120]. Collectively, these results suggest an outsized role for nurse bee nutrient processing in larval dietary macronutrients, and an overall loss of lipids comparing source pollen to processed food. In honey bees, nurses and foragers are distinct task specialists that show extensive physiological differences [121,122]. Previous research has shown that nurse bees often either do not seem to show nutrition-based preferences for particular pollens or show distinct preferences from foragers, which could contribute some to the difference between forager input (foragers choose the pollen to bring to the hive) and nurse output (nurses consume a portion of this pollen and generate jellies) [114,123,124]. However, nurse bee physiological needs may also play an important role in the macronutrient ratio discrepancy comparing pollen to jellies. In the first few days following adult emergence, worker honey bees increase their stored fat, the majority of which accrues in the multipurpose fat body organ [125]. This lipid assimilation prepares the worker to perform nursing behaviors [126], and lipid consumption during the entire nursing timeframe (~days 3–12 of adult life) is important for development and maintenance of the hypopharyngeal glands used to generate worker jelly [127–131]. Our data and that from other studies of worker jelly and pollen suggest that a significant amount of the lipids that nurses consume appear to be retained rather than converted into worker jelly, which suggests they do not typically contribute lipids from their own fat stores to worker jelly under normal nutritional conditions [127,132,133]. This could explain why older workers are able to perform nursing behaviors even with very low fat stores [122,134]. Proteins could show opposite patterns, however, since they show increased concentration in worker jelly relative to source pollen. Although minimum dietary protein is critical for nurses to generate the protein in jelly [135], there is also evidence that exposure to social cues that regulate nurse care activities mobilize abdominal protein stores for use in larval jelly production [136]. This suggests nurses have the capacity to buffer low protein intake, and they perhaps routinely supplement dietary protein intake with their own nutritional stores. Further work is needed to understand the potential for nurse bees to protect larvae from variation in floral resource availability—this ability may be nutrient-specific. Importantly, this buffering capacity (or lack thereof) makes honey bee resource demands distinct from most other bees where larvae eat pollen directly [137].

We designed our study to assess whether variation in colony aggression level predicted the quantity or makeup of worker jelly. Similar study approaches revealed that colony aggression levels experienced during larval development affect adult defensive aggression and immune system function for workers [41,48], and that nurses from high-aggression colonies show different care responses to alarm pheromone [54]. In all of these studies, including the current study, variation in aggression likely originated from a combination of genetic and environmental sources, and all colonies were European-derived genetic strains from diverse sources. While aggression—and associated variation in adult worker behavior and physiology [41,45,48,138–140]—did not explain significant variation in worker jelly quantity and content in the current study, among-colony variance was greater for high-aggression colonies than low-aggression colonies. This result reinforces the fact that diverse factors drive up aggression levels, and that variation in aggression could have variable relationships to brood provisioning (including no relationship). Our previous finding, that nurse bees are more likely to decrease nursing effort during alarm pheromone exposure in high-aggression colonies, might suggest that high-aggression colonies have variable or less predictable food availability over time [54,141], a characteristic that can engender physiological resilience later in life [142,143] and may explain some positive health outcomes associated with aggression [45,48,144]. An alternative interpretation of our results is that within-colony variance was more substantial in low-aggression colonies compared to high-aggression colonies, and this reduced our ability to detect among-colony variation. Developmental environments characterized by low aggression have been associated with a stressed immune phenotype in emerging adult honey bees [48]; perhaps the low-aggression colonies struggle to provide consistent nutrition compared to high-aggression colonies. Note that the current study as well as the previously mentioned studies included only honey bees derived from European strains and did not include so-called “Africanized” bees (a hybrid between European- and African-derived sub-species). Although Africanized bees often show more extreme levels of defensive aggression than the bees in our study, we did not compare Africanized to European strains because they have other unique features of their biology that could have confounded our results, such as faster development, greater nest relocation frequency, and lower degrees of honey hoarding [145–148]. Our original goal was to explain environmentally induced variation in aggression (and immune system function) among siblings that developed in high- and low-aggression European honey bee colonies [41,48], but future studies could explicitly examine and compare jelly from Africanized sources.

As with many colony-level honey bee studies, we noted several unusual colonies in our data. For example, the highest colony-average total dry mass per sample (found in colony H9) is nearly five times higher than the lowest colony-average total dry mass per sample (colony H4). Additionally, one colony (H7) had a significantly higher proportion of mass in the “other” category—that is, mass that is not accounted for by proteins, lipids, or carbohydrates—than any other colony in this study. This latter phenomenon would likely not be due to technical error in the sample collection or measurement process, as all of H7’s samples were collected and weighed at the same time as another colony, L7, which showed typical amounts of “other” mass, and all samples were randomized between plates during the colorimetric assays. We do not have a definitive explanation for this result, nor do we know what comprises the “other” mass, though it could be anything from nondigestible fiber to contaminants [149–153]. This study was limited to three major macronutrients: proteins, lipids, and carbohydrates. Many other components are known to be present in worker jelly, such as phytochemicals, vitamins, minerals, other micronutrients, microorganisms, and bioactive compounds such as neurotransmitters and hormones [40,59,60,154]. Future work could assess the intra- and inter-colony variability of these smaller components, which may help explain some of the abnormalities noted in our experiment and could reveal closer associations with our traits of interest (e.g., aggression). Similarly, while this study compared whole protein content between samples, the exact protein composition could also vary on top of the total protein variability observed in this study, with potential implications for larval health [155,156]. Proteomics and/or metabolomics could reveal whether these more detailed measures vary more or less than total macronutrient measures [85,157].

Our study took the larval perspective and examined the food in a cell available to a larva at a single point in time, which could include additions from multiple nurses. Future studies could seek to determine the level of individual variation from the perspective of nurse bees by extracting worker jelly from individual nurses and comparing it with that of their colony mates. Similarly, our study—which focused on 2-day-old larvae—cannot say whether other larval stages would show more, less, or a similar amount of variation associated with our measured variables. We also cannot rule out the possibility that the larvae influenced the food we collected them on. Honey bee larvae do not defecate until right before pupation, so we do not expect excretory contamination [56]. Likewise, the authors are not aware of any study documenting honey bee larvae regurgitating and re-consuming jelly (as some insects do, e.g., [57]), so we do not expect larval modification in this regard. But it is possible that within- or among-colony variation in the rate of jelly consumption exists. Such variation, should it exist, could have enhanced the observed variation in total dry mass we observed. The data on worker jelly consumption rates is somewhat limited. Most studies have been done in-vitro with artificial food or with queen larvae, and colony-level replication is lacking. The data that do exist do not provide evidence that differences in larval consumption rate are likely to be high enough to have been a major factor. For example, Dietz and Lambremont measured twelve worker larvae’s jelly consumption rates on artificial diet. At the same age as our measured cohort, the coefficient of variation of the consumption rates was only 0.15, markedly less than the coefficients of variation we observed in total dry massof our larval jelly samples [158].

Additionally, we did not measure individual or colony-level outcomes for the larvae in our study. It is possible that the significant natural within- and among-colony variation we observed would not be enough to cause effects at the colony scale. However, potential outcomes based on previous experimental work are diverse and range in subtlety. Larval food deprivation is associated with higher rates of developmental failure during the pupal stage and changes in body weight and morphometrics [159,160]. Larvae from colonies with an experimentally reduced nursing workforce show a ~ 25% decrease in adult lifespan and altered morphometrics relative to those with sufficient nursing effort [161,162]. Even minor underfeeding of larvae (much lower than the range of variation we observed in our study) can lead to reduced adult body size [163]. Queen/worker determination could also be affected; diet quantity can influence the development of queen traits, even beyond macronutrient composition [164]. Furthermore, larvae artificially reared on diets with non-plant-based protein sources show relatively normal body weights and survival rates but have altered behaviors and hormone profiles in adulthood [165,166]. Variation in carbohydrates (also observed in our study but to a lesser degree than proteins) in lab-based larval diets affects queen/worker caste determination [167]. In-vitro-reared larvae also show more variable ovariole numbers and altered physiological and hormonal measures compared to colony-reared larvae [168,169]. Together, these results suggest the possibility for wide-ranging fitness consequences for the nutritional variation detected in our study. Worker body size variation has implications for colony foraging efficiency [170]. Development failure and lifespan decreases would lead to a decrease in the total adult workforce, which affects everything from foraging effectiveness to future brood production capacity [171]. And alterations to queen/worker determination could have difficult-to-predict effects on everything from colony function to direct fitness consequences [172]. Thus, it seems likely that the degree of variation in worker jelly quantity and content we observed is sufficient to contribute to adult phenotypic variation, and by extension, colony health.

Theoretical and experimental work suggests that parental care can buffer against environmental variability and risk on an evolutionary scale [173,174]. The role of within-species variation in these dynamics, particularly in social species with cooperative brood care, provides an exciting new avenue for studying the developmental, physiological, behavioral, and health consequences of the early-life period.

## Supporting information

S1 Fig Coefficients of variation for each colony for each macronutrient. (PNG)

S2 Fig Ranges of macronutrients on a per-colony basis. Each line extends from the minimum to maximum value for each colony for A) total proteins, B) total lipids, and C) total carbohydrates.
(PNG)

S3 Fig Quantitative aggression score as a function of binomial aggression level. Boxplots show the distribution (following the convention in Figure 3) of raw aggression scores for colonies that were binned into the “high aggression” and “low aggression” categories; each dot represents one colony’s score. The raw aggression score was calculated as the difference between the number of bees at the colony entrance after an alarm pheromone presentation versus at baseline (see METHODS for details).
(PNG)

S4 Fig Colony-level aggression was not predictive of worker jelly nutritional content. Linear mixed models of each nutrient with site as a fixed effect and colony ID as a random effect all showed no significant differences. Boxplots of A) total dry mass, B) total protein mass, C) relative proteins, D) total lipid mass, E) relative lipids, F) total carbohydrate mass, and G) relative carbohydrates of worker jelly samples for high-aggression (red) and low-aggression (blue) colonies.
(PNG)

S5 Fig Scatterplots of total dry mass (mg) and relative quantities of different macronutrients in worker jelly as a function of colony aggression rank. Each dot represents an individual sample, black lines indicate the line of best fit (assuming a linear model), separated by aggression level. Grey shading around lines indicates the 95% confidence interval. Aggression rank (x-axis) is centered around zero, where “Low Aggression” colonies are negative numbers and “High Aggression” colonies are positive. Panels indicate A) total dry mass (mg), B) relative proteins, C) relative lipids, and D) relative carbohydrates.
(PNG)

S6 Fig Ranked aggression score is not predictive of worker jelly nutritional profiles. Scatterplots of aggression ranks (centered around zero) versus A) total dry mass, B) total protein mass, C) total lipid mass, and D) total carbohydrate mass of honey bee worker jelly samples. Note the use of total masses of proteins, lipids, and carbohydrates in this figure compared to in-text figures; relative proteins, lipids, and carbohydrates were qualitatively similar. Colors are indicative of how colonies were grouped into the high- versus low-aggression categories in the previous analysis where aggression was treated as a binomial variable.
(PNG)

S7 Fig Continuous aggression score is not predictive of any of the nutrients we measured. Scatterplots of raw aggression score versus A) total dry mass, B) total protein mass, C) total lipid mass, and D) total carbohydrate mass of honey bee worker jelly samples. Note the use of total masses of proteins, lipids, and carbohydrates in this figure compared to in-text figures; relative proteins, lipids, and carbohydrates were qualitatively similar. Colors are indicative of how colonies were grouped into high- versus low-aggression colonies in the previous analysis.
(PNG)

S8 Fig There is no clear seasonal trend over the course of the 23 days that samples were collected. All charts show the average mass in mg per nutrient by experimental day (with the first collection day being Day 1), separated by colony aggression level. A) Total dry mass (mg), B) relative protein quantity, C) relative lipid quantity, D) relative carbohydrate quantity.
(PNG)

S9 Fig Total dry mass and relative quantities of each macronutrient (total nutrient mass divided by total dry mass for each sample) as a function of colony site. Boxplots of A) total dry mass, B) relative proteins, C) relative lipids, and D) relative carbohydrates of worker jelly samples from three sites, Alpha (yellow), Beta (green), and Gamma (blue). Sites Alpha and Beta were approximately 1.6 km apart, while Gamma was approximately 14.5 km away from the other two sites. All comparisons are statistically nonsignificant based on linear mixed models.
(PNG)

S10 Fig Total dry mass and relative quantities of each macronutrient (total nutrient mass divided by total dry mass for each sample) as a function of colony site. Boxplots of A) total dry mass, B) relative proteins, C) relative lipids, and D) relative carbohydrates of worker jelly samples from colonies headed by queens from three genetic strains, Italian (purple), Russian Hybrid (red), and open-mated (yellow). All comparisons are statistically nonsignificant based on linear mixed models.
(PNG)

S11 Table Table listing Colony ID, colony aggression level, genetic strain, site of the colony, and the date the worker jelly was collected. (CSV)

S12 Table Comparison of published studies examining the nutritional content of worker jelly. Proteins, lipids, and carbohydrates are reported as a mean percentage of the dry mass per sample (± standard deviation, where reported). Larval age (h) indicates the time post-egg hatching. This is typically a range because queens are given a range of time to lay eggs (see METHODS). In many cases, macronutrients per sample were simply reported as a mean. “Other” indicates the percentage of dry mass not accounted for by the three macronutrients (e.g., micronutrients, nondigestible material, etc.), and this is listed as a sample mean or a range depending on how results were reported in the study. “NR” indicates the information was not reported by the study. Note that these studies use a constellation of different methodologies that likely enhances the variation.
(CSV)

S13 Table Raw Data. Excel file with all raw data used to generate this manuscript. This file has five sheets: the first sheet is a ReadMe, while the second through fifth sheets contain the four data files needed to generate all figures and statistical results reported in this study.
(XLSX)

S14 Code PDF of an RMarkdown file displaying all code used to generate the figures and statistical results used in this study. (PDF)
