# Supplementary figures and images for: Substantial variation in larval honey bee nutrition within and among Apis mellifera colonies
Source: PLoS One. 2026 Feb 25;21(2):e0328027. doi: 10.1371/journal.pone.0328027 (PMC12935248; doi:10.1371/journal.pone.0328027)

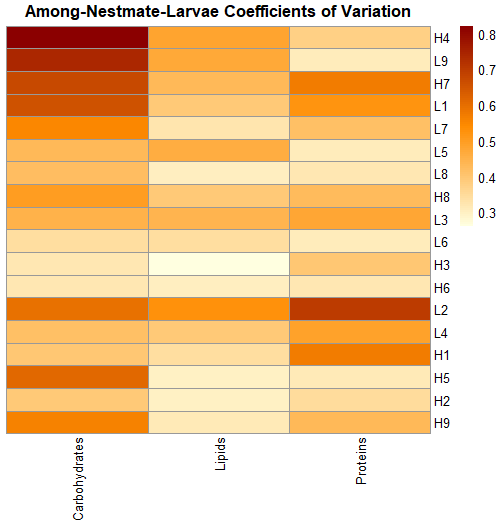

Supplement: S1 Fig — (PNG) [file pone.0328027.s001.png]

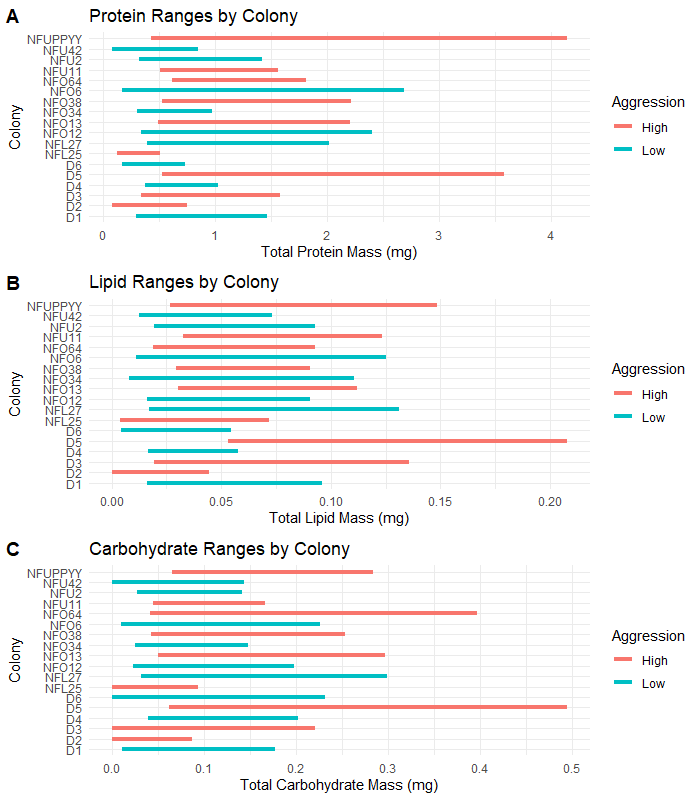

Supplement: S2 Fig — Each line extends from the minimum to maximum value for each colony for A) total proteins, B) total lipids, and C) total carbohydrates. (PNG) [file pone.0328027.s002.png]

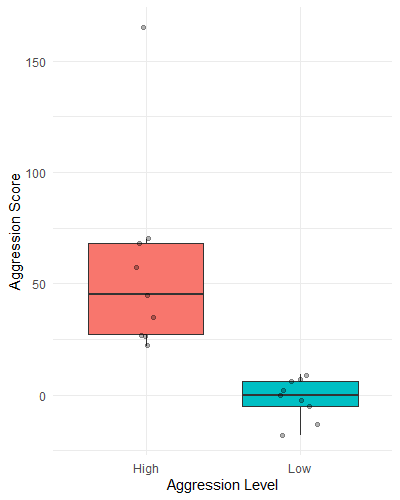

Supplement: S3 Fig — Boxplots show the distribution (following the convention in Figure 3) of raw aggression scores for colonies that were binned into the “high aggression” and “low aggression” categories; each dot represents one colony’s score. The raw aggression score was calculated as the difference between the number of bees at the colony entrance after an alarm pheromone presentation versus at baseline (see METHODS for details). (PNG) [file pone.0328027.s003.png]

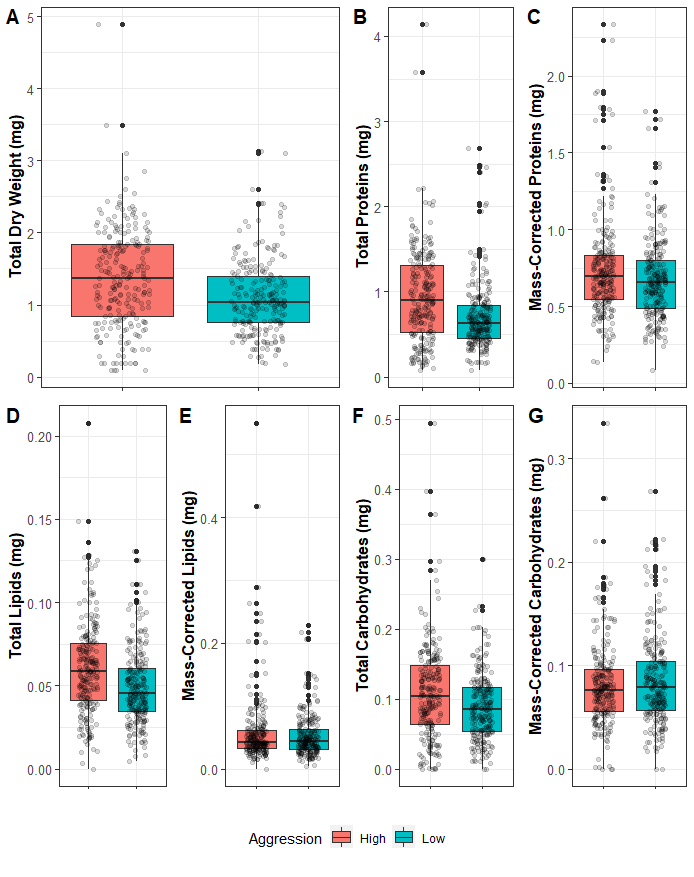

Supplement: S4 Fig — Linear mixed models of each nutrient with site as a fixed effect and colony ID as a random effect all showed no significant differences. Boxplots of A) total dry mass, B) total protein mass, C) relative proteins, D) total lipid mass, E) relative lipids, F) total carbohydrate mass, and G) relative carbohydrates of worker jelly samples for high-aggression (red) and low-aggression (blue) colonies. (PNG) [file pone.0328027.s004.png]

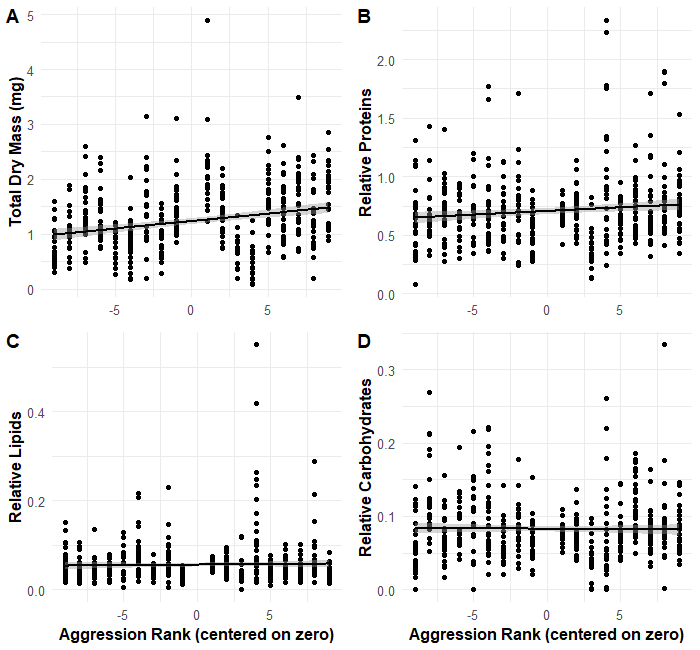

Supplement: S5 Fig — Each dot represents an individual sample, black lines indicate the line of best fit (assuming a linear model), separated by aggression level. Grey shading around lines indicates the 95% confidence interval. Aggression rank (x-axis) is centered around zero, where “Low Aggression” colonies are negative numbers and “High Aggression” colonies are positive. Panels indicate A) total dry mass (mg), B) relative proteins, C) relative lipids, and D) relative carbohydrates. (PNG) [file pone.0328027.s005.png]

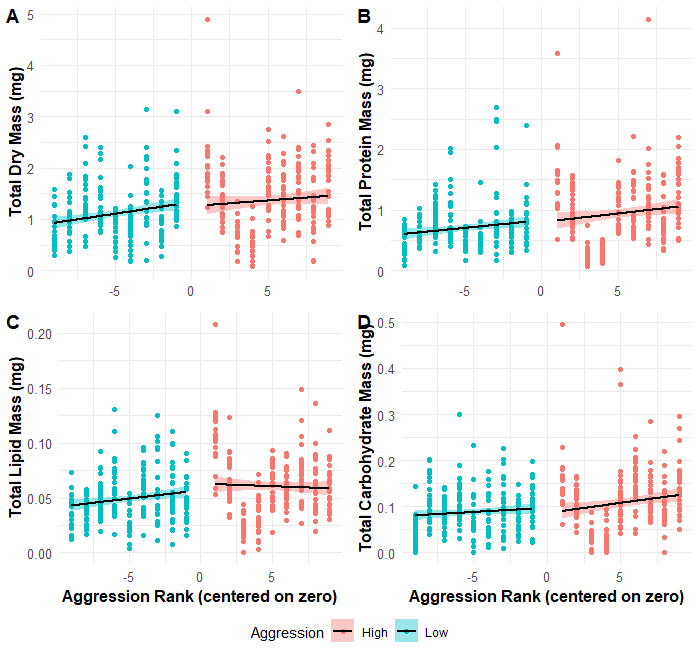

Supplement: S6 Fig — Scatterplots of aggression ranks (centered around zero) versus A) total dry mass, B) total protein mass, C) total lipid mass, and D) total carbohydrate mass of honey bee worker jelly samples. Note the use of total masses of proteins, lipids, and carbohydrates in this figure compared to in-text figures; relative proteins, lipids, and carbohydrates were qualitatively similar. Colors are indicative of how colonies were grouped into the high- versus low-aggression categories in the previous analysis where aggression was treated as a binomial variable. (PNG) [file pone.0328027.s006.png]

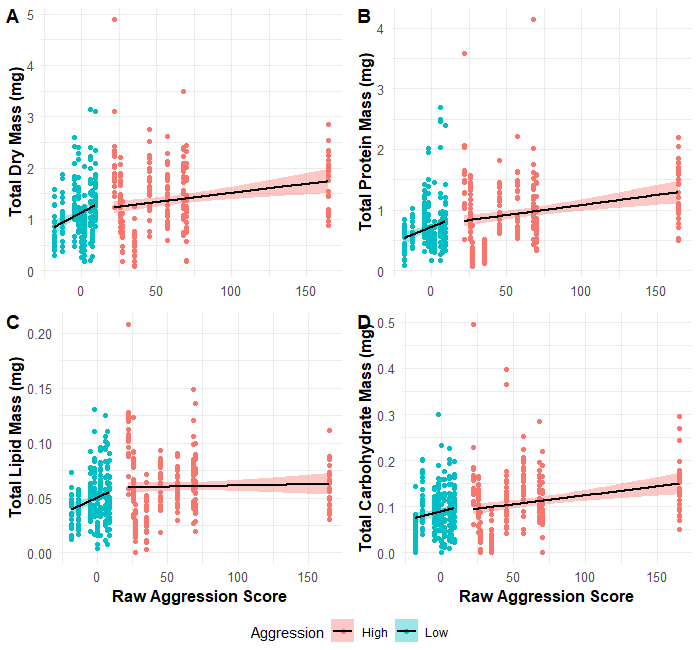

Supplement: S7 Fig — Scatterplots of raw aggression score versus A) total dry mass, B) total protein mass, C) total lipid mass, and D) total carbohydrate mass of honey bee worker jelly samples. Note the use of total masses of proteins, lipids, and carbohydrates in this figure compared to in-text figures; relative proteins, lipids, and carbohydrates were qualitatively similar. Colors are indicative of how colonies were grouped into high- versus low-aggression colonies in the previous analysis. (PNG) [file pone.0328027.s007.png]

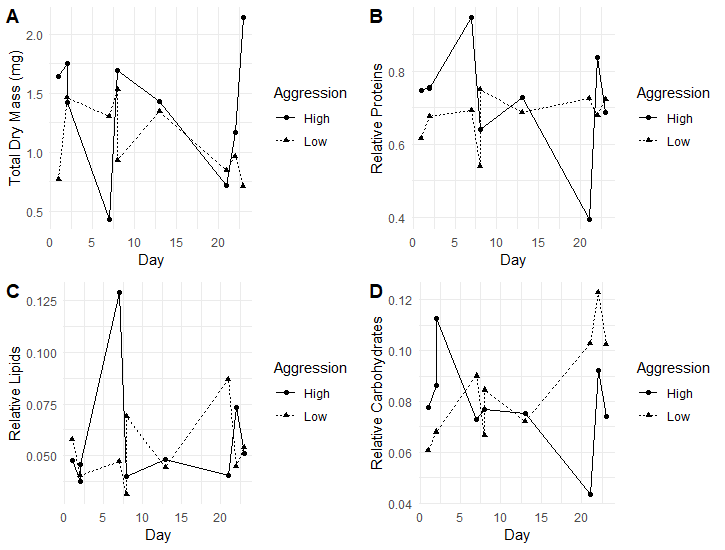

Supplement: S8 Fig — All charts show the average mass in mg per nutrient by experimental day (with the first collection day being Day 1), separated by colony aggression level. A) Total dry mass (mg), B) relative protein quantity, C) relative lipid quantity, D) relative carbohydrate quantity. (PNG) [file pone.0328027.s008.png]

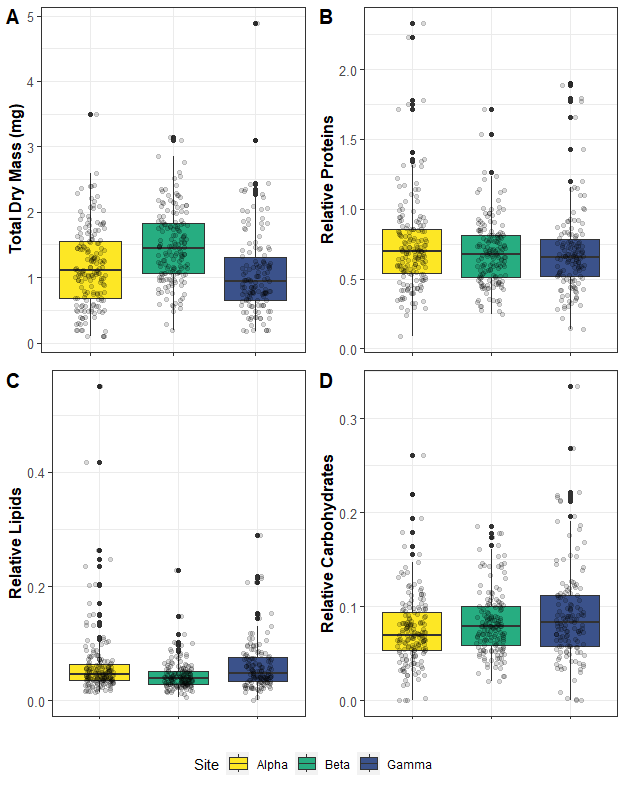

Supplement: S9 Fig — Boxplots of A) total dry mass, B) relative proteins, C) relative lipids, and D) relative carbohydrates of worker jelly samples from three sites, Alpha (yellow), Beta (green), and Gamma (blue). Sites Alpha and Beta were approximately 1.6 km apart, while Gamma was approximately 14.5 km away from the other two sites. All comparisons are statistically nonsignificant based on linear mixed models. (PNG) [file pone.0328027.s009.png]

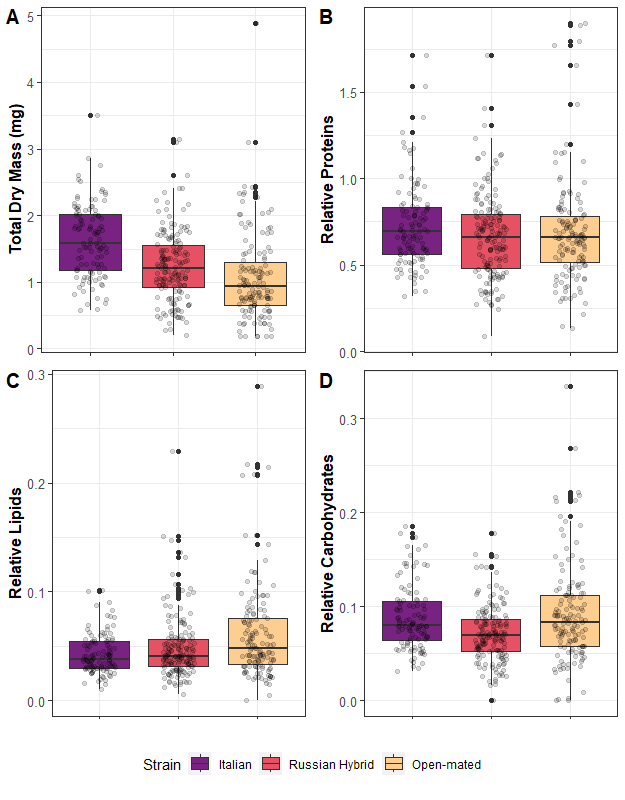

Supplement: S10 Fig — Boxplots of A) total dry mass, B) relative proteins, C) relative lipids, and D) relative carbohydrates of worker jelly samples from colonies headed by queens from three genetic strains, Italian (purple), Russian Hybrid (red), and open-mated (yellow). All comparisons are statistically nonsignificant based on linear mixed models. (PNG) [file pone.0328027.s010.png]
