# Supplementary material for: Substantial variation in larval honey bee nutrition within and among Apis mellifera colonies
Source: PLoS One. 2026 Feb 25;21(2):e0328027. doi: 10.1371/journal.pone.0328027 (PMC12935248; doi:10.1371/journal.pone.0328027)
Supplement: S14 Code — (PDF) [file pone.0328027.s014.pdf]

WorkerJelly2025_RCode

[Redacted for Peer Review]

12/18/2025

This code was used to generate figures and statistical results for the study, "Substantial variation in larval honey bee nutrition within and among Apis mellifera colonies."

All code was run on R version 4.1.2

Set working directory

```
setwd("C:/Users/Public/Brood Food Data")
```

Importing data

```
WorkerJelly2025_Data <- read.csv("WorkerJelly2025_Data.csv")
```

```
ColonyAggression <- read.csv("ColonyAggression.csv")
```

```
BFCV <- read.csv("BFCV.csv")
```

```
BF_Ranges <- read.csv("BF_Ranges.csv")
```

Install and load packages

```
install.packages("ggpubr", repos = "http://cran.us.r-project.org")
```

```
## Installing package into 'C:/Users/rw247/AppData/Local/R/win-library/4.5'
## (as 'lib' is unspecified)
```

```
## package 'ggpubr' successfully unpacked and MD5 sums checked
##
## The downloaded binary packages are in
##  C:\Users\rw247\AppData\Local\Temp\Rtmpem3WMp\downloaded_packages
```

```r
install.packages("ggbiplot", repos = "http://cran.us.r-project.org")
```

```
## Installing package into 'C:/Users/rw247/AppData/Local/R/win-library/4.5'
## (as 'lib' is unspecified)

## package 'ggbiplot' successfully unpacked and MD5 sums checked
##
## The downloaded binary packages are in
##  C:\Users\rw247\AppData\Local\Temp\Rtmpem3WMp\downloaded_packages
```

```r
install.packages("lme4", repos = "http://cran.us.r-project.org")
```

```
## Installing package into 'C:/Users/rw247/AppData/Local/R/win-library/4.5'
## (as 'lib' is unspecified)

## package 'lme4' successfully unpacked and MD5 sums checked
##
## The downloaded binary packages are in
##  C:\Users\rw247\AppData\Local\Temp\Rtmpem3WMp\downloaded_packages
```

```r
install.packages("DHARMa", repos = "http://cran.us.r-project.org")
```

```
## Installing package into 'C:/Users/rw247/AppData/Local/R/win-library/4.5'
## (as 'lib' is unspecified)

## package 'DHARMa' successfully unpacked and MD5 sums checked
##
## The downloaded binary packages are in
##  C:\Users\rw247\AppData\Local\Temp\Rtmpem3WMp\downloaded_packages
```

```r
install.packages("car", repos = "http://cran.us.r-project.org")
```

```
## Installing package into 'C:/Users/rw247/AppData/Local/R/win-library/4.5'
## (as 'lib' is unspecified)

## package 'car' successfully unpacked and MD5 sums checked
##
## The downloaded binary packages are in
##  C:\Users\rw247\AppData\Local\Temp\Rtmpem3WMp\downloaded_packages
```

```r
install.packages("ggplot2", repos = "http://cran.us.r-project.org")
```

```
## Installing package into 'C:/Users/rw247/AppData/Local/R/win-library/4.5'
## (as 'lib' is unspecified)

## package 'ggplot2' successfully unpacked and MD5 sums checked
##
## The downloaded binary packages are in
##  C:\Users\rw247\AppData\Local\Temp\Rtmpem3WMp\downloaded_packages
```

```r
install.packages("cowplot", repos = "http://cran.us.r-project.org")
```

```
## Installing package into 'C:/Users/rw247/AppData/Local/R/win-library/4.5'
## (as 'lib' is unspecified)
```

```
## package 'cowplot' successfully unpacked and MD5 sums checked
##
## The downloaded binary packages are in
##  C:\Users\rw247\AppData\Local\Temp\Rtmpem3WMp\downloaded_packages
```

```r
install.packages("viridis", repos = "http://cran.us.r-project.org")
```

```
## Installing package into 'C:/Users/rw247/AppData/Local/R/win-library/4.5'
## (as 'lib' is unspecified)
```

```
## package 'viridis' successfully unpacked and MD5 sums checked
##
## The downloaded binary packages are in
##  C:\Users\rw247\AppData\Local\Temp\Rtmpem3WMp\downloaded_packages
```

```r
install.packages("reshape2", repos = "http://cran.us.r-project.org")
```

```
## Installing package into 'C:/Users/rw247/AppData/Local/R/win-library/4.5'
## (as 'lib' is unspecified)
```

```
## package 'reshape2' successfully unpacked and MD5 sums checked
##
## The downloaded binary packages are in
##  C:\Users\rw247\AppData\Local\Temp\Rtmpem3WMp\downloaded_packages
```

```r
install.packages("pheatmap", repos = "http://cran.us.r-project.org")
```

```
## Installing package into 'C:/Users/rw247/AppData/Local/R/win-library/4.5'
## (as 'lib' is unspecified)
```

```
## package 'pheatmap' successfully unpacked and MD5 sums checked
##
## The downloaded binary packages are in
##  C:\Users\rw247\AppData\Local\Temp\Rtmpem3WMp\downloaded_packages
```

```r
library(ggpubr)
```

```
## Warning: package 'ggpubr' was built under R version 4.5.2
```

```
## Loading required package: ggplot2
```

```
## Warning: package 'ggplot2' was built under R version 4.5.2
```

```r
library(ggbiplot)
```

```
## Warning: package 'ggbiplot' was built under R version 4.5.2
```

```r
library(lme4)
```

```
## Warning: package 'lme4' was built under R version 4.5.2
```

```
## Loading required package: Matrix
```

```r
library(DHARMa)
```

```
## Warning: package 'DHARMa' was built under R version 4.5.2
```

```
## This is DHARMa 0.4.7. For overview type '?DHARMa'. For recent changes, type news(package = 'DHARMa')
```

```r
library(car)
```

```
## Warning: package 'car' was built under R version 4.5.2
```

```
## Loading required package: carData
```

```r
library(ggplot2)
```

```r
library(cowplot)
```

```
## Warning: package 'cowplot' was built under R version 4.5.2
```

```
##
## Attaching package: 'cowplot'
```

```
## The following object is masked from 'package:ggpubr':
##
##     get_legend
```

```r
library(viridis)
```

```
## Warning: package 'viridis' was built under R version 4.5.2
```

```
## Loading required package: viridisLite
```

```r
library(reshape2)
```

```
## Warning: package 'reshape2' was built under R version 4.5.2
```

```r
library(pheatmap)
```

```
## Warning: package 'pheatmap' was built under R version 4.5.2
```

Calculating means and standard deviations of total dry mass and mass of each macronutrient

Total dry mass

```r
mean(WorkerJelly2025_Data$TotalDryWeight)
```

```
## [1] 1.237116
```

```r
sd(WorkerJelly2025_Data$TotalDryWeight)
```

```
## [1] 0.6299095
```

Total proteins

```r
mean(WorkerJelly2025_Data$TotalProtein)
```

```
## [1] 0.8234034
```

```r
sd(WorkerJelly2025_Data$TotalProtein)
```

```
## [1] 0.4970442
```

Total lipids

```r
mean(WorkerJelly2025_Data$TotalLipids)
```

```
## [1] 0.05470486
```

```r
sd(WorkerJelly2025_Data$TotalLipids)
```

```
## [1] 0.02613098
```

Total carbohydratess

```r
mean(WorkerJelly2025_Data$TotalCarbs)
```

```
## [1] 0.09834593
```

```r
sd(WorkerJelly2025_Data$TotalCarbs)
```

```
## [1] 0.05998735
```

Generating and plotting Principle Components Analysis of both total masses and relative quantities of proteins, lipids, and carbohydrates (Figure 1)

```r
ggarrange(ggbiplot(prcomp(WorkerJelly2025_Data[, c(14, 16, 18)],
    center = TRUE, scale. = TRUE), ellipse = TRUE, groups = WorkerJelly2025_Data$Aggression,
    alpha = 0.33) + theme_minimal() + theme(legend.position = "none"),
    ggbiplot(prcomp(WorkerJelly2025_Data[, c(13, 15, 17)], center = TRUE,
        scale. = TRUE), ellipse = TRUE, groups = WorkerJelly2025_Data$Aggression,
        alpha = 0.33) + guides(color = guide_legend("Aggression"),
        fill = guide_legend("Aggression")) + coord_cartesian(clip = "off") +
        theme_minimal(), labels = c("A", "B"), ncol = 2, nrow = 1,
    widths = c(1.6, 2))
```

```
## Coordinate system already present.
## i Adding new coordinate system, which will replace the existing one.
```

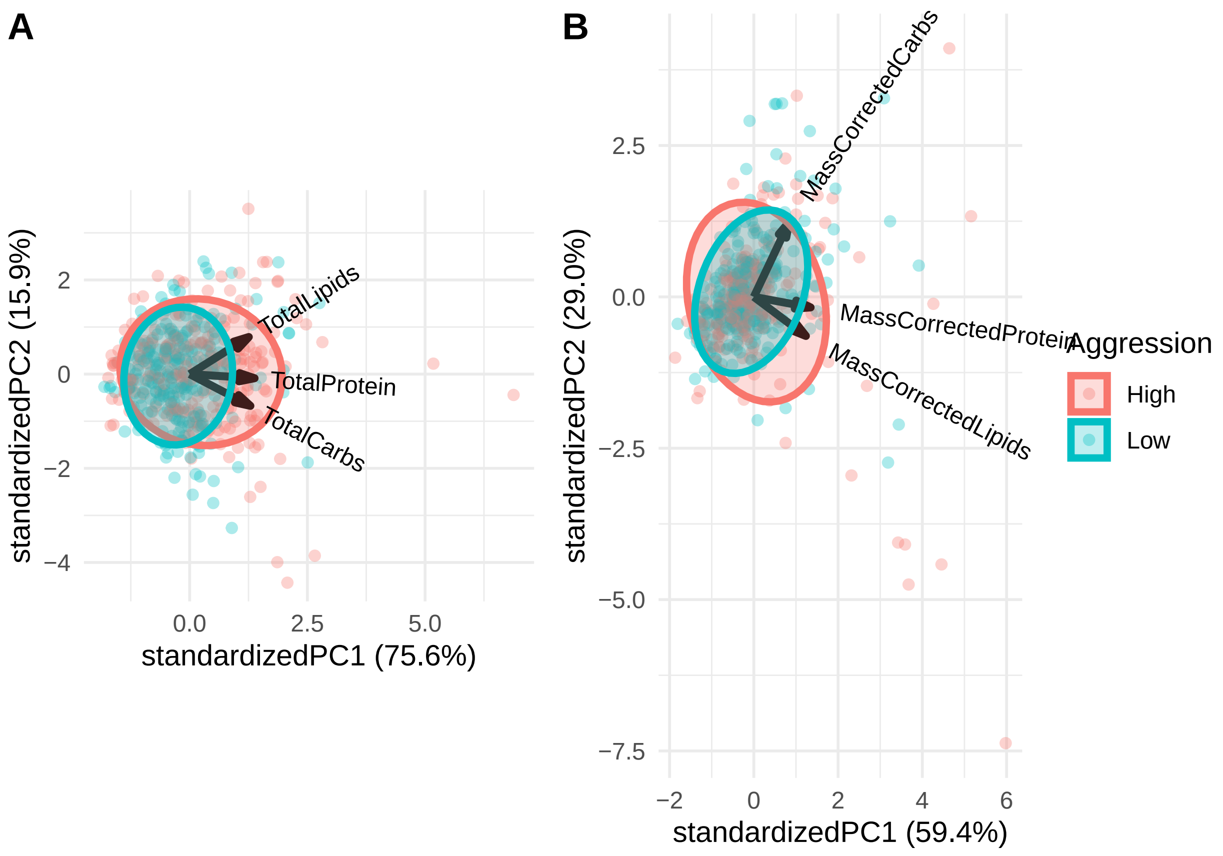

Calculating colony-level means of total dry mass and each macronutrient total mass

Creating a new dataframe with colony-level means

```
NutrientMeans <- data.frame(aggregate(WorkerJelly2025_Data$TotalDryWeight,
    list(WorkerJelly2025_Data$Hive, WorkerJelly2025_Data$Day),
    FUN = mean), aggregate(WorkerJelly2025_Data$TotalProtein,
    list(WorkerJelly2025_Data$Hive, WorkerJelly2025_Data$Day),
    FUN = mean), aggregate(WorkerJelly2025_Data$TotalLipids,
    list(WorkerJelly2025_Data$Hive, WorkerJelly2025_Data$Day),
    FUN = mean), aggregate(WorkerJelly2025_Data$TotalCarbs, list(WorkerJelly2025_Data$Hive,
    WorkerJelly2025_Data$Day), FUN = mean))
```

Removing extra columns

```
NutrientMeans = subset(NutrientMeans, select = -c(Group.1.1,
    Group.2.1, Group.1.2, Group.2.2, Group.1.3, Group.2.3))
```

Renaming columns

```
colnames(NutrientMeans) <- c("Hive", "Day", "AvgTDW", "AvgTP",
    "AvgTL", "AvgTC")
```

Modeling among-nestmate versus among-colony variation

Checking model assumptions

From here on, *all* linear mixed models had their assumptions checked with the DHARMa package to assess model fit and determine the best data transformations. Here is a sample of how that code would look. We are only noting this code once for space and repetition reasons. The user can substitute in any of the models in the rest of the document to see the diagnostics for that particular model.

```
plot(simulateResiduals(lm(log(TotalDryWeight + 1) ~ Hive, data = WorkerJelly2025_Data)))
```

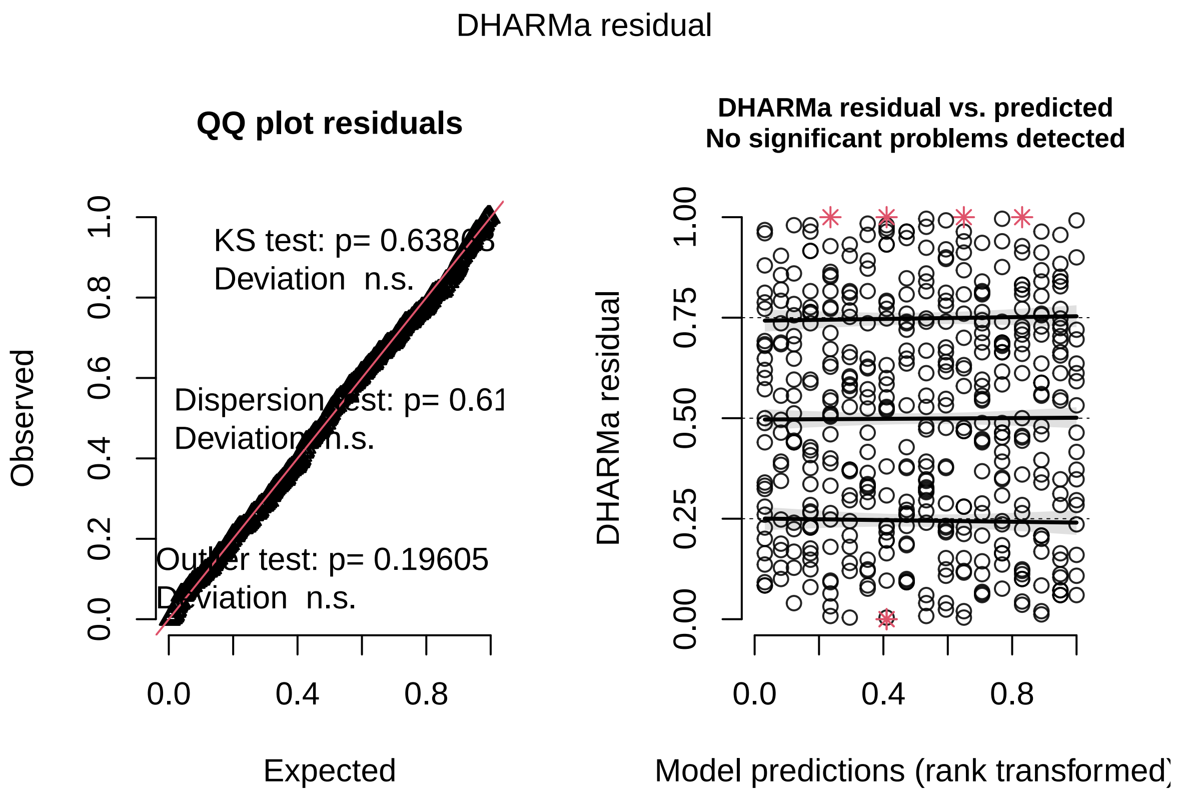

Running the model and obtaining significance values

For total dry mass

```r
Anova(lm(sqrt(TotalDryWeight) ~ Hive, data = WorkerJelly2025_Data))
```

```
## Anova Table (Type II tests)
##
## Response: sqrt(TotalDryWeight)
##           Sum Sq  Df F value    Pr(>F)
## Hive       18.92  17  24.388 < 2.2e-16 ***
## Residuals  20.49 449
## ---
## Signif. codes:  0 '***' 0.001 '**' 0.01 '*' 0.05 '.' 0.1 ' ' 1
```

For total proteins

```r
Anova(lm(sqrt(TotalProtein) ~ Hive, data = WorkerJelly2025_Data))
```

```
## Anova Table (Type II tests)
##
## Response: sqrt(TotalProtein)
##           Sum Sq  Df F value    Pr(>F)
## Hive      15.495  17  26.105 < 2.2e-16 ***
## Residuals 15.677 449
## ---
## Signif. codes:  0 '***' 0.001 '**' 0.01 '*' 0.05 '.' 0.1 ' ' 1
```

For total lipids

```r
Anova(lm(sqrt(TotalLipids) ~ Hive, data = WorkerJelly2025_Data))
```

```
## Anova Table (Type II tests)
##
## Response: sqrt(TotalLipids)
##           Sum Sq  Df F value    Pr(>F)
## Hive      0.5834  17  17.255 < 2.2e-16 ***
## Residuals 0.8930 449
## ---
## Signif. codes:  0 '***' 0.001 '**' 0.01 '*' 0.05 '.' 0.1 ' ' 1
```

For total carbohydrates

```r
Anova(lm(sqrt(TotalCarbs) ~ Hive, data = WorkerJelly2025_Data))
```

```
## Anova Table (Type II tests)
##
## Response: sqrt(TotalCarbs)
##           Sum Sq  Df F value    Pr(>F)
```

```
## Hive      1.9987  17   20.07 < 2.2e-16 ***
## Residuals 2.6303 449
## ---
## Signif. codes:  0 '***' 0.001 '**' 0.01 '*' 0.05 '.' 0.1 ' ' 1
```

Testing for homogeneity of variance

For total dry mass

```r
leveneTest(sqrt(TotalDryWeight) ~ Hive, data = WorkerJelly2025_Data)
```

```
## Warning in leveneTest.default(y = y, group = group, ...): group coerced to
## factor.

## Levene's Test for Homogeneity of Variance (center = median)
##        Df F value Pr(>F)
## group  17  0.8809 0.5972
##       449
```

For total proteins

```r
leveneTest(sqrt(TotalProtein) ~ Hive, data = WorkerJelly2025_Data)
```

```
## Warning in leveneTest.default(y = y, group = group, ...): group coerced to
## factor.

## Levene's Test for Homogeneity of Variance (center = median)
##        Df F value   Pr(>F)
## group  17  2.7591 0.000216 ***
##       449
## ---
## Signif. codes:  0 '***' 0.001 '**' 0.01 '*' 0.05 '.' 0.1 ' ' 1
```

For total lipids

```r
leveneTest(sqrt(TotalLipids) ~ Hive, data = WorkerJelly2025_Data)
```

```
## Warning in leveneTest.default(y = y, group = group, ...): group coerced to
## factor.

## Levene's Test for Homogeneity of Variance (center = median)
##        Df F value Pr(>F)
## group  17  1.3607 0.1516
##       449
```

For total carbohydrates

```r
leveneTest(sqrt(TotalCarbs) ~ Hive, data = WorkerJelly2025_Data)
```

```
## Warning in leveneTest.default(y = y, group = group, ...): group coerced to
## factor.
```

```
## Levene's Test for Homogeneity of Variance (center = median)
##        Df F value Pr(>F)
## group  17  0.9137 0.5578
##       449
```

Running models to assess the association between colony aggression and nutrition

For total dry mass

```r
Anova(lmer(log(TotalDryWeight + 1) ~ Aggression + (1 | Hive),
    data = WorkerJelly2025_Data))
```

```
## Analysis of Deviance Table (Type II Wald chisquare tests)
##
## Response: log(TotalDryWeight + 1)
##             Chisq Df Pr(>Chisq)
## Aggression 1.3068  1      0.253
```

For relative proteins

```r
Anova(lmer(sqrt(MassCorrectedProtein) ~ Aggression + (1 | Hive),
    data = WorkerJelly2025_Data))
```

```
## Analysis of Deviance Table (Type II Wald chisquare tests)
##
## Response: sqrt(MassCorrectedProtein)
##            Chisq Df Pr(>Chisq)
## Aggression 0.644  1     0.4223
```

For relative lipids

```r
Anova(lmer(sqrt(MassCorrectedLipids) ~ Aggression + (1 | Hive),
    data = WorkerJelly2025_Data))
```

```
## Analysis of Deviance Table (Type II Wald chisquare tests)
##
## Response: sqrt(MassCorrectedLipids)
##             Chisq Df Pr(>Chisq)
## Aggression 0.0684  1     0.7937
```

For relative carbohydrates

```
Anova(lmer(sqrt(MassCorrectedCarbs) ~ Aggression + (1 | Hive),
    data = WorkerJelly2025_Data))
```

```
## Analysis of Deviance Table (Type II Wald chisquare tests)
##
## Response: sqrt(MassCorrectedCarbs)
##             Chisq Df Pr(>Chisq)
## Aggression 0.4247  1     0.5146
```

Assessing among-nestmate versus among-colony variance within each aggression level

Creating a dataframe of only high-aggression colonies

```
WorkerJelly2025_Data_High <- subset(WorkerJelly2025_Data, Aggression ==
    "High")
```

Creating a dataframe of only low-aggression colonies

```
WorkerJelly2025_Data_Low <- subset(WorkerJelly2025_Data, Aggression ==
    "Low")
```

Total dry mass, high aggression

```
summary(aov(sqrt(TotalDryWeight) ~ Hive, data = WorkerJelly2025_Data_High))
```

```
##              Df Sum Sq Mean Sq F value Pr(>F)
## Hive          8  13.12  1.6393   33.58 <2e-16 ***
## Residuals   225  10.98  0.0488
## ---
## Signif. codes:  0 '***' 0.001 '**' 0.01 '*' 0.05 '.' 0.1 ' ' 1
```

Total dry mass, low aggression

```
summary(aov(sqrt(TotalDryWeight) ~ Hive, data = WorkerJelly2025_Data_Low))
```

```
##              Df Sum Sq Mean Sq F value   Pr(>F)
## Hive          8  4.579  0.5723   13.49 6.75e-16 ***
## Residuals   224  9.506  0.0424
## ---
## Signif. codes:  0 '***' 0.001 '**' 0.01 '*' 0.05 '.' 0.1 ' ' 1
```

Total proteins, high aggression

```
summary(aov(sqrt(TotalProtein) ~ Hive, data = WorkerJelly2025_Data_High))
```

```
##                Df Sum Sq Mean Sq F value Pr(>F)
## Hive            8 11.076  1.3844   38.14 <2e-16 ***
## Residuals     225  8.167  0.0363
## ---
## Signif. codes:  0 '***' 0.001 '**' 0.01 '*' 0.05 '.' 0.1 ' ' 1
```

Total proteins, low aggression

```
summary(aov(sqrt(TotalProtein) ~ Hive, data = WorkerJelly2025_Data_Low))
```

```
##                Df Sum Sq Mean Sq F value   Pr(>F)
## Hive            8  2.782  0.3477   10.37 2.47e-12 ***
## Residuals     224  7.510  0.0335
## ---
## Signif. codes:  0 '***' 0.001 '**' 0.01 '*' 0.05 '.' 0.1 ' ' 1
```

Total lipids, high aggression

```
summary(aov(sqrt(TotalLipids) ~ Hive, data = WorkerJelly2025_Data_High))
```

```
##                Df Sum Sq Mean Sq F value Pr(>F)
## Hive            8 0.4131 0.05163   28.37 <2e-16 ***
## Residuals     225 0.4095 0.00182
## ---
## Signif. codes:  0 '***' 0.001 '**' 0.01 '*' 0.05 '.' 0.1 ' ' 1
```

Total lipids, low aggression

```
summary(aov(sqrt(TotalLipids) ~ Hive, data = WorkerJelly2025_Data_Low))
```

```
##                Df Sum Sq  Mean Sq F value   Pr(>F)
## Hive            8 0.1116 0.013946   6.461 1.46e-07 ***
## Residuals     224 0.4835 0.002158
## ---
## Signif. codes:  0 '***' 0.001 '**' 0.01 '*' 0.05 '.' 0.1 ' ' 1
```

Total carbohydrates, high aggression

```
summary(aov(sqrt(TotalCarbs) ~ Hive, data = WorkerJelly2025_Data_High))
```

```
##              Df Sum Sq Mean Sq F value Pr(>F)
## Hive          8  1.564 0.19552    33.5 <2e-16 ***
## Residuals   225  1.313 0.00584
## ---
## Signif. codes:  0 '***' 0.001 '**' 0.01 '*' 0.05 '.' 0.1 ' ' 1
```

Total carbohydrates, low aggression

```
summary(aov(sqrt(TotalCarbs) ~ Hive, data = WorkerJelly2025_Data_Low))
```

```
##              Df Sum Sq Mean Sq F value   Pr(>F)
## Hive          8 0.3532 0.04415   7.508 7.19e-09 ***
## Residuals   224 1.3171 0.00588
## ---
## Signif. codes:  0 '***' 0.001 '**' 0.01 '*' 0.05 '.' 0.1 ' ' 1
```

Assessing the effects of temporal variation

For total dry mass

```
Anova(lmer(sqrt(TotalDryWeight) ~ Day + (1 | Hive), data = WorkerJelly2025_Data))
```

```
## Analysis of Deviance Table (Type II Wald chisquare tests)
##
## Response: sqrt(TotalDryWeight)
##      Chisq Df Pr(>Chisq)
## Day 0.8081  1     0.3687
```

For relative proteins

```
Anova(lmer(sqrt(MassCorrectedProtein) ~ Day + (1 | Hive), data = WorkerJelly2025_Data))
```

```
## Analysis of Deviance Table (Type II Wald chisquare tests)
##
## Response: sqrt(MassCorrectedProtein)
##      Chisq Df Pr(>Chisq)
## Day 0.2541  1     0.6142
```

For relative lipids

```r
Anova(lmer(sqrt(MassCorrectedLipids) ~ Day + (1 | Hive), data = WorkerJelly2025_Data))
```

```
## Analysis of Deviance Table (Type II Wald chisquare tests)
##
## Response: sqrt(MassCorrectedLipids)
##      Chisq Df Pr(>Chisq)
## Day 0.3328  1      0.564
```

For relative carbohydrates

```r
Anova(lmer(sqrt(MassCorrectedCarbs) ~ Day + (1 | Hive), data = WorkerJelly2025_Data))
```

```
## Analysis of Deviance Table (Type II Wald chisquare tests)
##
## Response: sqrt(MassCorrectedCarbs)
##      Chisq Df Pr(>Chisq)
## Day 0.4214  1     0.5162
```

Running models to assess an alternative hypothesis, association between site (location of the colony) and nutrition

For total dry mass

```r
Anova(lmer(log(TotalDryWeight + 1) ~ Location + (1 | Hive), data = WorkerJelly2025_Data))
```

```
## Analysis of Deviance Table (Type II Wald chisquare tests)
##
## Response: log(TotalDryWeight + 1)
##           Chisq Df Pr(>Chisq)
## Location 2.8814  2     0.2368
```

For relative proteins

```r
Anova(lmer(sqrt(MassCorrectedProtein) ~ Location + (1 | Hive),
    data = WorkerJelly2025_Data))
```

```
## Analysis of Deviance Table (Type II Wald chisquare tests)
##
## Response: sqrt(MassCorrectedProtein)
##           Chisq Df Pr(>Chisq)
## Location 0.7241  2     0.6962
```

For relative lipids

```
Anova(lmer(sqrt(MassCorrectedLipids) ~ Location + (1 | Hive),
    data = WorkerJelly2025_Data))
```

```
## Analysis of Deviance Table (Type II Wald chisquare tests)
##
## Response: sqrt(MassCorrectedLipids)
##           Chisq Df Pr(>Chisq)
## Location 2.5706  2     0.2766
```

For relative carbohydrates

```
Anova(lmer(sqrt(MassCorrectedCarbs) ~ Location + (1 | Hive),
    data = WorkerJelly2025_Data))
```

```
## Analysis of Deviance Table (Type II Wald chisquare tests)
##
## Response: sqrt(MassCorrectedCarbs)
##           Chisq Df Pr(>Chisq)
## Location 1.3895  2     0.4992
```

Running models to assess a second alternative hypothesis, association between colony genetic strain (here called "source") and nutrition

Creating a dataframe that only includes samples from colonies with sufficient replication

```
WorkerJelly2025_Data_Source <- subset(WorkerJelly2025_Data, Source ==
    "Italian" | Source == "RussianHybrid" | Source == "Wild stock",
    select = c(Hive:TotalCarbs))
```

Now running the models

For total dry mass

```
Anova(lmer(log(TotalDryWeight + 1) ~ Source + (1 | Hive), data = WorkerJelly2025_Data_Source))
```

```
## Analysis of Deviance Table (Type II Wald chisquare tests)
##
## Response: log(TotalDryWeight + 1)
##         Chisq Df Pr(>Chisq)
## Source 5.5637  2    0.06192 .
## ---
## Signif. codes:  0 '***' 0.001 '**' 0.01 '*' 0.05 '.' 0.1 ' ' 1
```

For relative proteins

```r
Anova(lmer(sqrt(MassCorrectedProtein) ~ Source + (1 | Hive),
    data = WorkerJelly2025_Data_Source))
```

```
## Analysis of Deviance Table (Type II Wald chisquare tests)
##
## Response: sqrt(MassCorrectedProtein)
##         Chisq Df Pr(>Chisq)
## Source 0.9055  2     0.6359
```

For relative lipids

```r
Anova(lmer(sqrt(MassCorrectedLipids) ~ Source + (1 | Hive), data = WorkerJelly2025_Data_Source))
```

```
## Analysis of Deviance Table (Type II Wald chisquare tests)
##
## Response: sqrt(MassCorrectedLipids)
##         Chisq Df Pr(>Chisq)
## Source 2.5108  2      0.285
```

For relative carbohydrates

```r
Anova(lmer(sqrt(MassCorrectedCarbs) ~ Source + (1 | Hive), data = WorkerJelly2025_Data_Source))
```

```
## Analysis of Deviance Table (Type II Wald chisquare tests)
##
## Response: sqrt(MassCorrectedCarbs)
##         Chisq Df Pr(>Chisq)
## Source 2.4756  2       0.29
```

Testing whether aggression level and strain are collinear

For total dry mass

```r
vif(lmer((TotalDryWeight) ~ Aggression + Source + (1 | Hive),
    data = WorkerJelly2025_Data_Source))
```

```
##                GVIF Df GVIF^(1/(2*Df))
## Aggression 1.719382  1        1.311252
## Source     1.719382  2        1.145099
```

For relative proteins

```r
vif(lmer(sqrt(MassCorrectedProtein) ~ Aggression + Source + (1 |
    Hive), data = WorkerJelly2025_Data_Source))
```

```
##                GVIF Df GVIF^(1/(2*Df))
## Aggression 1.725879  1        1.313727
## Source     1.725879  2        1.146179
```

For relative lipids

```r
vif(lmer(sqrt(MassCorrectedLipids) ~ Aggression + Source + (1 |
    Hive), data = WorkerJelly2025_Data_Source))
```

```
##                GVIF Df GVIF^(1/(2*Df))
## Aggression 1.725274  1        1.313497
## Source     1.725274  2        1.146079
```

For relative carbohydrates

```r
vif(lmer(sqrt(MassCorrectedCarbs) ~ Aggression + Source + (1 |
    Hive), data = WorkerJelly2025_Data_Source))
```

```
##                GVIF Df GVIF^(1/(2*Df))
## Aggression 1.724698  1        1.313278
## Source     1.724698  2        1.145983
```

Testing for an interaction effect between aggression and strain

For total dry mass

```r
Anova(lmer(log(TotalDryWeight + 1) ~ Aggression * Source + (1 |
    Hive), data = WorkerJelly2025_Data_Source), type = "III")
```

```
## fixed-effect model matrix is rank deficient so dropping 1 column / coefficient
```

```
## Analysis of Deviance Table (Type III Wald chisquare tests)
##
## Response: log(TotalDryWeight + 1)
##                      Chisq Df Pr(>Chisq)
## (Intercept)       137.2282  1     <2e-16 ***
## Aggression          2.3595  1     0.1245
## Source              1.4758  2     0.4781
## Aggression:Source   0.1714  1     0.6789
## ---
## Signif. codes:  0 '***' 0.001 '**' 0.01 '*' 0.05 '.' 0.1 ' ' 1
```

For relative proteins

```
Anova(lmer(sqrt(MassCorrectedProtein) ~ Aggression * Source +
    (1 | Hive), data = WorkerJelly2025_Data_Source), type = "III")
```

```
## fixed-effect model matrix is rank deficient so dropping 1 column / coefficient
```

```
## Analysis of Deviance Table (Type III Wald chisquare tests)
##
## Response: sqrt(MassCorrectedProtein)
##                       Chisq Df Pr(>Chisq)
## (Intercept)        577.8318  1     <2e-16 ***
## Aggression           0.6471  1     0.4212
## Source               1.3557  2     0.5077
## Aggression:Source    1.1325  1     0.2873
## ---
## Signif. codes:  0 '***' 0.001 '**' 0.01 '*' 0.05 '.' 0.1 ' ' 1
```

For relative lipids

```
Anova(lmer(sqrt(MassCorrectedLipids) ~ Aggression * Source +
    (1 | Hive), data = WorkerJelly2025_Data_Source), type = "III")
```

```
## fixed-effect model matrix is rank deficient so dropping 1 column / coefficient
```

```
## Analysis of Deviance Table (Type III Wald chisquare tests)
##
## Response: sqrt(MassCorrectedLipids)
##                       Chisq Df Pr(>Chisq)
## (Intercept)        183.9597  1     <2e-16 ***
## Aggression           0.2541  1     0.6142
## Source               0.8780  2     0.6447
## Aggression:Source    0.1867  1     0.6657
## ---
## Signif. codes:  0 '***' 0.001 '**' 0.01 '*' 0.05 '.' 0.1 ' ' 1
```

For relative carbohydrates

```
Anova(lmer(sqrt(TotalCarbs) ~ Aggression * Source + (1 | Hive),
    data = WorkerJelly2025_Data_Source), type = "III")
```

```
## fixed-effect model matrix is rank deficient so dropping 1 column / coefficient
```

```
## Analysis of Deviance Table (Type III Wald chisquare tests)
##
## Response: sqrt(TotalCarbs)
##                       Chisq Df Pr(>Chisq)
```

```
## (Intercept)       157.5269  1     <2e-16 ***
## Aggression          0.0015  1     0.9690
## Source              3.0821  2     0.2142
## Aggression:Source   0.2952  1     0.5869
## ---
## Signif. codes:  0 '***' 0.001 '**' 0.01 '*' 0.05 '.' 0.1 ' ' 1
```

Creating Figures

Figure 1 already shown above

Figure 2, bar plots of total masses and relative quantities of each macronutrient

Creating long dataframe

```r
WorkerJelly2025_Data$Other <- (WorkerJelly2025_Data$TotalDryWeight -
    WorkerJelly2025_Data$TotalProtein - WorkerJelly2025_Data$TotalLipids -
    WorkerJelly2025_Data$TotalCarbs)
```

Creating "other" column (everything that's not proteins, lipids, or carbohydrates to make the row add up to the total dry mass)

```r
long_WorkerJelly2025_Data <- melt(WorkerJelly2025_Data, measure.vars = c("TotalProtein",
    "TotalLipids", "TotalCarbs", "Other"), variable.name = "Measurement",
    value.name = "Value")
```

Converting to long format

```r
long_WorkerJelly2025_Data$Measurement <- factor(long_WorkerJelly2025_Data$Measurement,
    levels = c("Other", "TotalCarbs", "TotalLipids", "TotalProtein"))
```

Making the "Measurement" column a factor with defined levels

Finally, creating the barplot

```r
ggarrange(ggplot(long_WorkerJelly2025_Data, aes(fill = Measurement,
    y = Value, x = reorder(Colony, Day))) + geom_bar(position = "stack",
    stat = "summary", fun = "mean") + facet_wrap(~Aggression,
    labeller = labeller(Aggression = c(High = "High Aggression",
        Low = "Low Aggression")), scales = "free_x") + scale_x_discrete(name = "Colony ID (ordered by da
    scale_fill_manual(values = c("gray68", "#d8c1f7", "#a185c9",
```

```
        "#74559e"), labels = c("Other Mass", "Total Carbohydrate Mass",
        "Total Lipid Mass", "Total Protein Mass")) + theme_minimal() +
    ylab("Mass of Nutrient (mg) (total height=total dry mass)") +
    theme(axis.title.x = element_blank()), ggplot(long_WorkerJelly2025_Data,
    aes(fill = Measurement, y = Value, x = reorder(Colony, Day))) +
    geom_bar(position = "fill", stat = "summary", fun = "mean") +
    facet_wrap(~Aggression, labeller = labeller(Aggression = c(High = "High Aggression",
        Low = "Low Aggression")), scales = "free_x") + scale_x_discrete(name = "Colony ID (ordered by da
    scale_fill_manual(values = c("gray68", "#d8c1f7", "#a185c9",
        "#74559e"), labels = c("Other Mass", "Relative Carbohydrates",
        "Relative Lipids", "Relative Proteins")) + theme_minimal() +
    ylab("Proportion of Total Dry Mass"), labels = c("A", "B"),
    ncol = 1, nrow = 2)
```

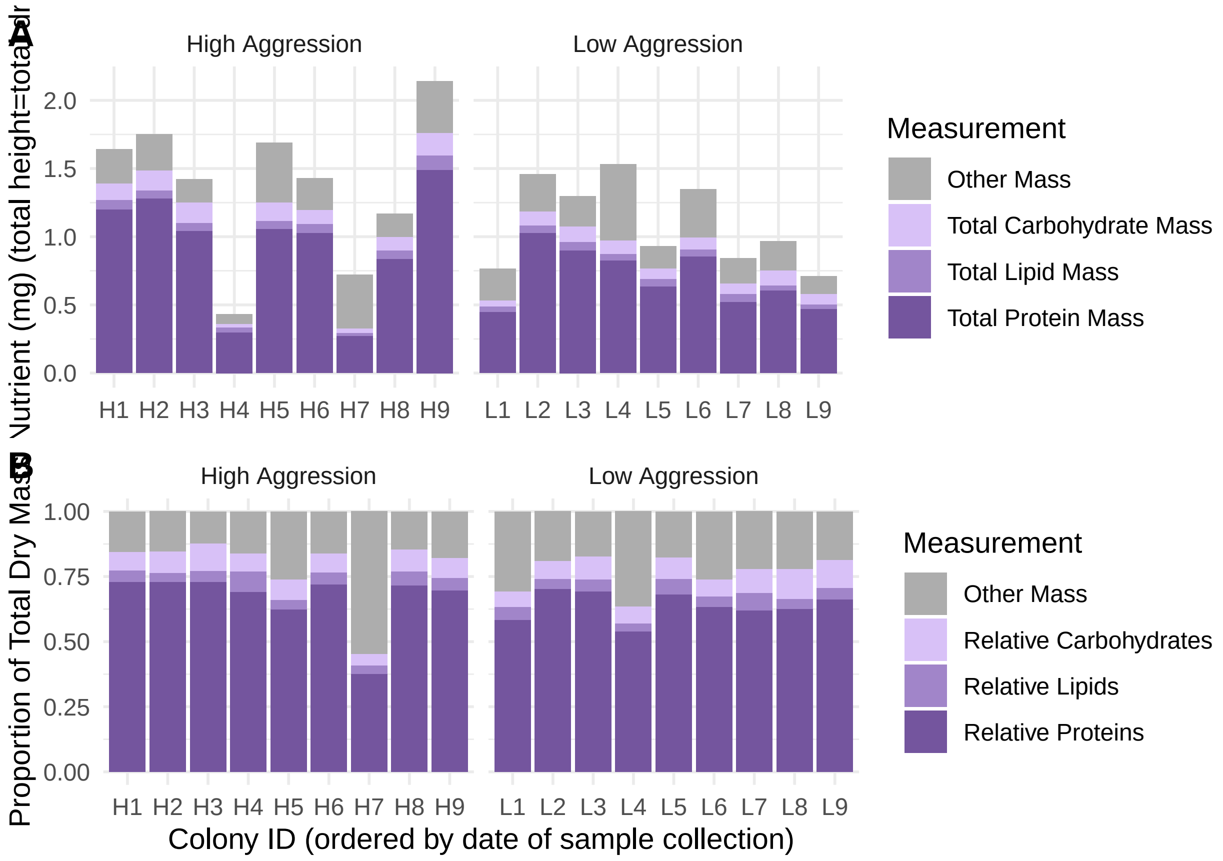

Figure 3, boxplots of total dry mass and relative macronutrient quantities, split by aggression level (I'm so sorry in advance, I know my code is very messy, but it works!)

Creating colony-level plots

```
DryMassPlot_Colony <- ggplot(WorkerJelly2025_Data, aes(x = reorder(Colony,
    -TotalDryWeight), y = TotalDryWeight, fill = Aggression)) +
    geom_boxplot() + geom_jitter(width = 0.15, alpha = 0.15) +
```

```
    labs(fill = "Aggression") + theme_minimal() + theme(strip.text = element_text(size = 14,
    face = "bold"), legend.position = "none", axis.text = element_text(size = 12),
    axis.title = element_blank())
```

```
ProteinPlot_Colony <- ggplot(WorkerJelly2025_Data, aes(x = reorder(Colony,
    -MassCorrectedProtein), y = MassCorrectedProtein, fill = Aggression)) +
    geom_boxplot() + geom_jitter(width = 0.15, alpha = 0.15) +
    labs(fill = "Aggression") + theme_minimal() + theme(strip.text = element_text(size = 14,
    face = "bold"), legend.position = "none", axis.text = element_text(size = 12),
    axis.title = element_blank())
```

```
LipidPlot_Colony <- ggplot(WorkerJelly2025_Data, aes(x = reorder(Colony,
    -MassCorrectedLipids), y = MassCorrectedLipids, fill = Aggression)) +
    geom_boxplot() + geom_jitter(width = 0.15, alpha = 0.15) +
    labs(fill = "Aggression") + theme_minimal() + theme(strip.text = element_text(size = 14,
    face = "bold"), legend.position = "none", axis.text = element_text(size = 12),
    axis.title = element_blank())
```

```
CarbPlot_Colony <- ggplot(WorkerJelly2025_Data, aes(x = reorder(Colony,
    -MassCorrectedCarbs), y = MassCorrectedCarbs, fill = Aggression)) +
    geom_boxplot() + geom_jitter(width = 0.15, alpha = 0.15) +
    xlab("Colony") + labs(fill = "Aggression") + theme_minimal() +
    theme(strip.text = element_text(size = 14, face = "bold"),
        legend.position = "none", axis.text = element_text(size = 12),
        axis.title = element_text(size = 14, face = "bold"),
        axis.title.y = element_blank())
```

###Putting it together with pooled plots

```
plot_grid(ggdraw() + draw_plot(ggplot(WorkerJelly2025_Data, aes(x = Aggression,
    y = TotalDryWeight, fill = Aggression)) + geom_boxplot() +
    geom_point(position = position_jitterdodge(jitter.width = 0.4),
        alpha = 0.15) + xlab("Aggression") + ylab("Total Dry Mass (mg)") +
    theme_bw() + theme(strip.text = element_text(size = 12, face = "bold"),
    axis.text = element_text(size = 10), axis.title = element_text(size = 10,
        face = "bold")) + theme(legend.position = "none", axis.text.x = element_blank(),
    axis.title.x = element_blank()), x = 0, y = 0.75, width = 0.25,
    height = 0.25) + draw_plot(DryMassPlot_Colony, x = 0.25,
    y = 0.75, width = 0.75, height = 0.25) + draw_plot(ggplot(WorkerJelly2025_Data,
    aes(x = Aggression, y = MassCorrectedProtein, fill = Aggression)) +
    geom_boxplot() + geom_point(position = position_jitterdodge(jitter.width = 0.4),
    alpha = 0.15) + xlab("Aggression") + ylab("Relative Proteins") +
    theme_bw() + theme(strip.text = element_text(size = 12, face = "bold"),
    axis.text = element_text(size = 10), axis.title = element_text(size = 10,
        face = "bold")) + theme(legend.position = "none", axis.text.x = element_blank(),
    axis.title.x = element_blank()), x = 0, y = 0.5, width = 0.25,
    height = 0.25) + draw_plot(ProteinPlot_Colony, x = 0.25,
    y = 0.5, width = 0.75, height = 0.25) + draw_plot(ggplot(WorkerJelly2025_Data,
    aes(x = Aggression, y = MassCorrectedLipids, fill = Aggression)) +
    geom_boxplot() + geom_point(position = position_jitterdodge(jitter.width = 0.4),
    alpha = 0.15) + xlab("Aggression") + ylab("Relative Lipids") +
    theme_bw() + theme(strip.text = element_text(size = 12, face = "bold"),
```

```
    axis.text = element_text(size = 10), axis.title = element_text(size = 10,
        face = "bold")) + theme(legend.position = "none", axis.text.x = element_blank(),
    axis.title.x = element_blank()), x = 0, y = 0.25, width = 0.25,
    height = 0.25) + draw_plot(LipidPlot_Colony, x = 0.25, y = 0.25,
    width = 0.75, height = 0.25) + draw_plot(ggplot(WorkerJelly2025_Data,
    aes(x = Aggression, y = MassCorrectedCarbs, fill = Aggression)) +
    geom_boxplot() + geom_point(position = position_jitterdodge(jitter.width = 0.4),
    alpha = 0.15) + xlab("Aggression") + ylab("Relative Carbohydrates") +
    theme_bw() + theme(strip.text = element_text(size = 12, face = "bold"),
    axis.text = element_text(size = 10), axis.title = element_text(size = 10,
        face = "bold")) + theme(legend.position = "none", axis.text.x = element_blank(),
    axis.title.x = element_blank()), x = 0, y = 0, width = 0.25,
    height = 0.25) + draw_plot(CarbPlot_Colony, x = 0.25, y = 0,
    width = 0.75, height = 0.25) + draw_plot_label(label = c("A",
    "B", "C", "D"), size = 15, x = c(0, 0, 0, 0), y = c(1, 0.75,
    0.5, 0.25)), get_legend((ggplot(WorkerJelly2025_Data, aes(x = Aggression,
    y = TotalDryWeight, fill = Aggression)) + geom_boxplot()) +
    guides(color = guide_legend(nrow = 1)) + theme(legend.position = "bottom")),
    ncol = 1, rel_heights = c(1, 0.1))
```

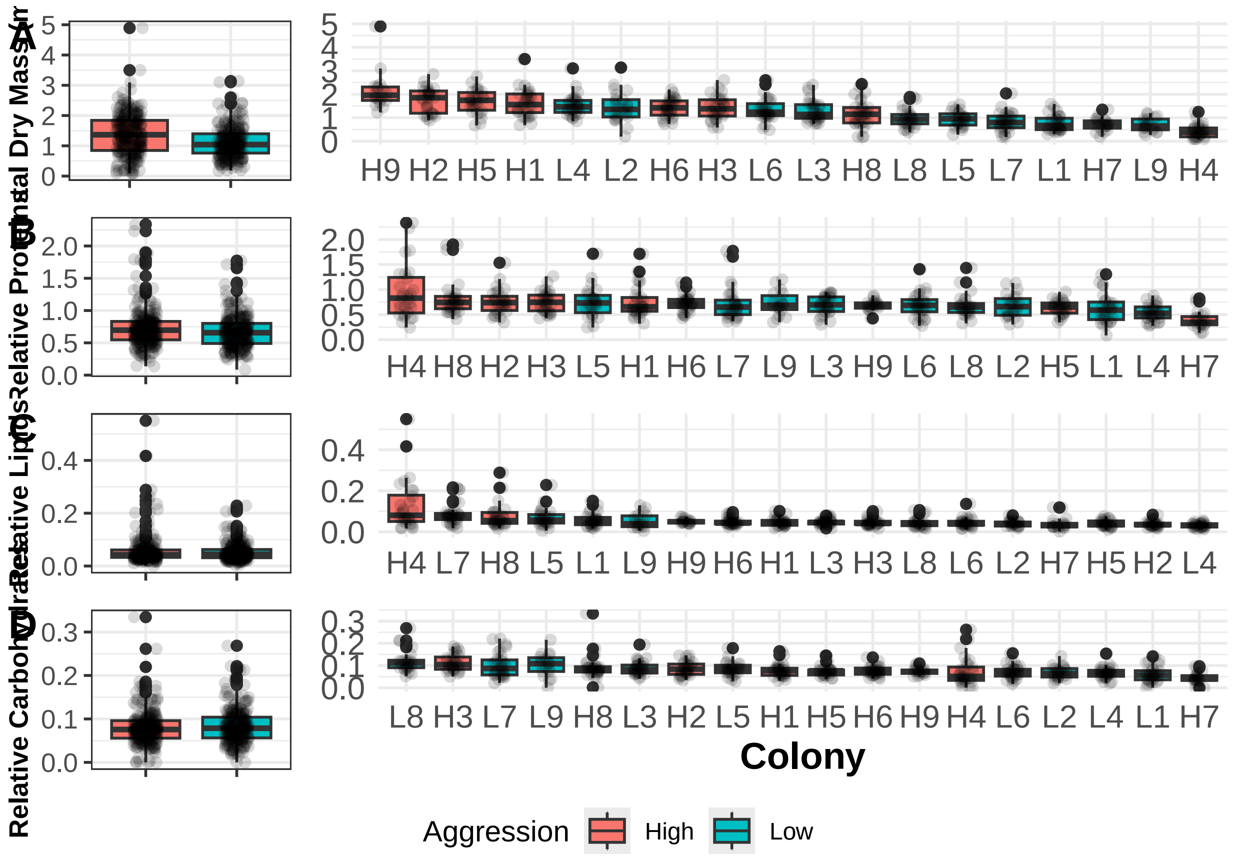

Creating Supplemental Data Figures

Figure S1, Heatmap of CVs

###Making the data a matrix

```
BFCVmat <- as.matrix(BFCV[, (2:4)])
```

###Fixing the row names

```
rownames(BFCVmat) <- (BFCV[, 1])
```

###Making the heatmap

```
pheatmap(BFCVmat, cluster_rows = FALSE, cluster_cols = FALSE,
    main = "Within-colony Coefficients of Variation", colorRampPalette(c("lightyellow",
        "darkorange", "darkred"))(100), angle_col = 90)
```

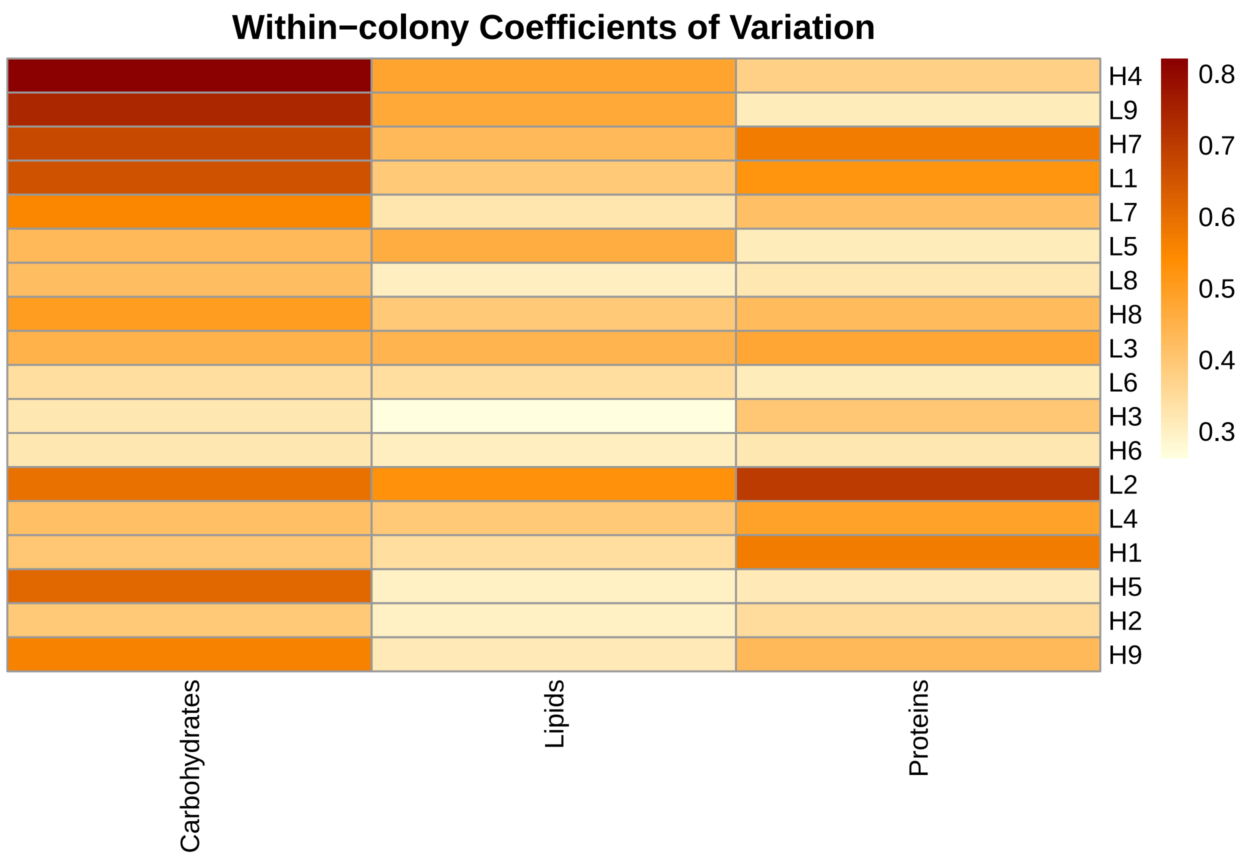

Figure S2, line plot of nutrient mass ranges

```
ggarrange(ggplot(BF_Ranges, aes(x = ProteinsMin, xend = ProteinsMax,
    y = Hive, color = Aggression)) + geom_segment(aes(xend = ProteinsMax),
```

```
    size = 1.5) + labs(title = "Protein Ranges by Colony", y = "Colony",
    x = "Total Protein Mass (mg)") + theme_minimal(), ggplot(BF_Ranges,
    aes(x = LipidsMin, xend = LipidsMax, y = Hive, color = Aggression)) +
    geom_segment(aes(xend = LipidsMax), size = 1.5) + labs(title = "Lipid Ranges by Colony",
    y = "Colony", x = "Total Lipid Mass (mg)") + theme_minimal(),
    ggplot(BF_Ranges, aes(x = CarbsMin, xend = CarbsMax, y = Hive,
        color = Aggression)) + geom_segment(aes(xend = CarbsMax),
        size = 1.5) + labs(title = "Carbohydrate Ranges by Colony",
        y = "Colony", x = "Total Carbohydrate Mass (mg)") + theme_minimal(),
    labels = c("A", "B", "C"), ncol = 1, nrow = 3)
```

```
## Warning: Using ‘size‘ aesthetic for lines was deprecated in ggplot2 3.4.0.
## i Please use ‘linewidth‘ instead.
## This warning is displayed once every 8 hours.
## Call ‘lifecycle::last_lifecycle_warnings()‘ to see where this warning was
## generated.
```

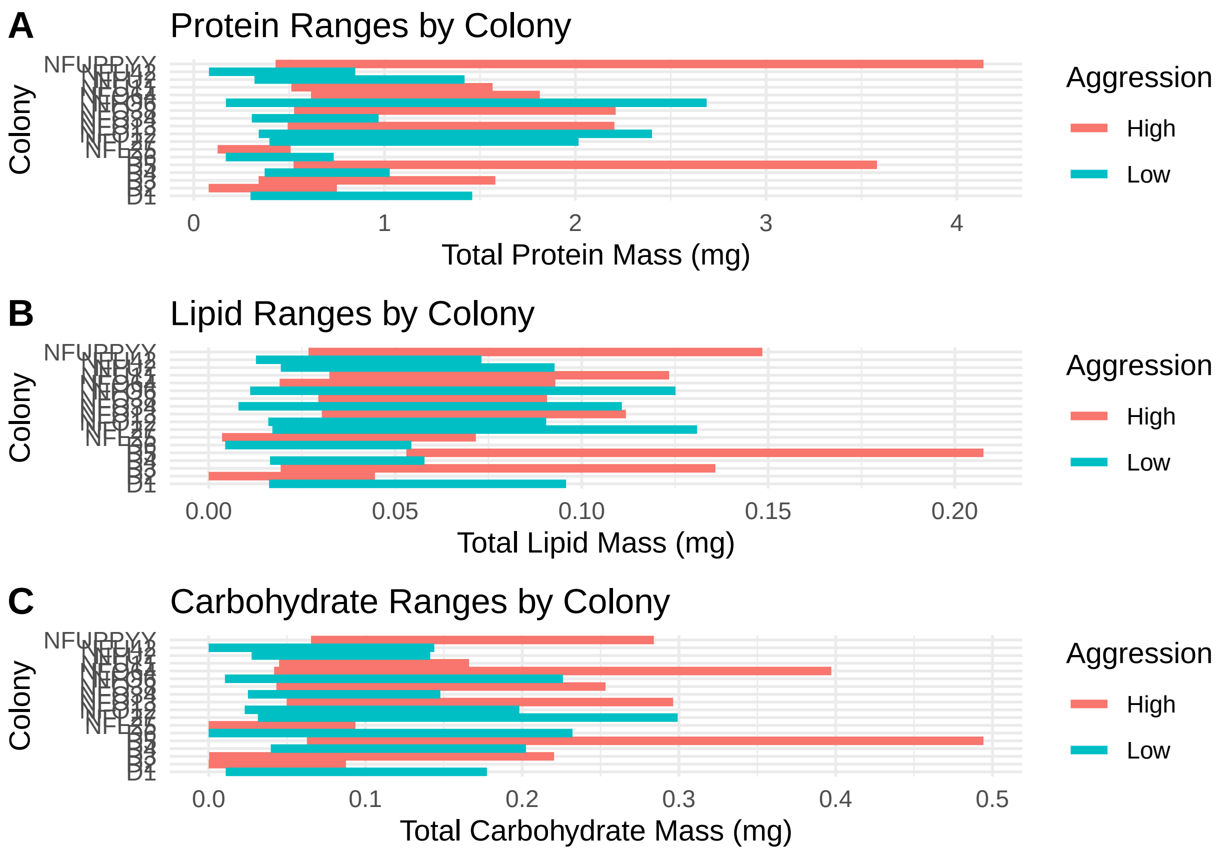

Figure S3, boxplot of quantitative aggression scores; uses the "ColonyAggression" dataset

```
ggplot(ColonyAggression, aes(fill = Aggression, y = AggScore,
    x = Aggression)) + geom_boxplot(outlier.shape = NA) + geom_jitter(alpha = 0.3,
```

```
    width = 0.15) + ylab("Aggression Score") + xlab("Aggression Level") +
    theme_minimal() + theme(legend.position = "none")
```

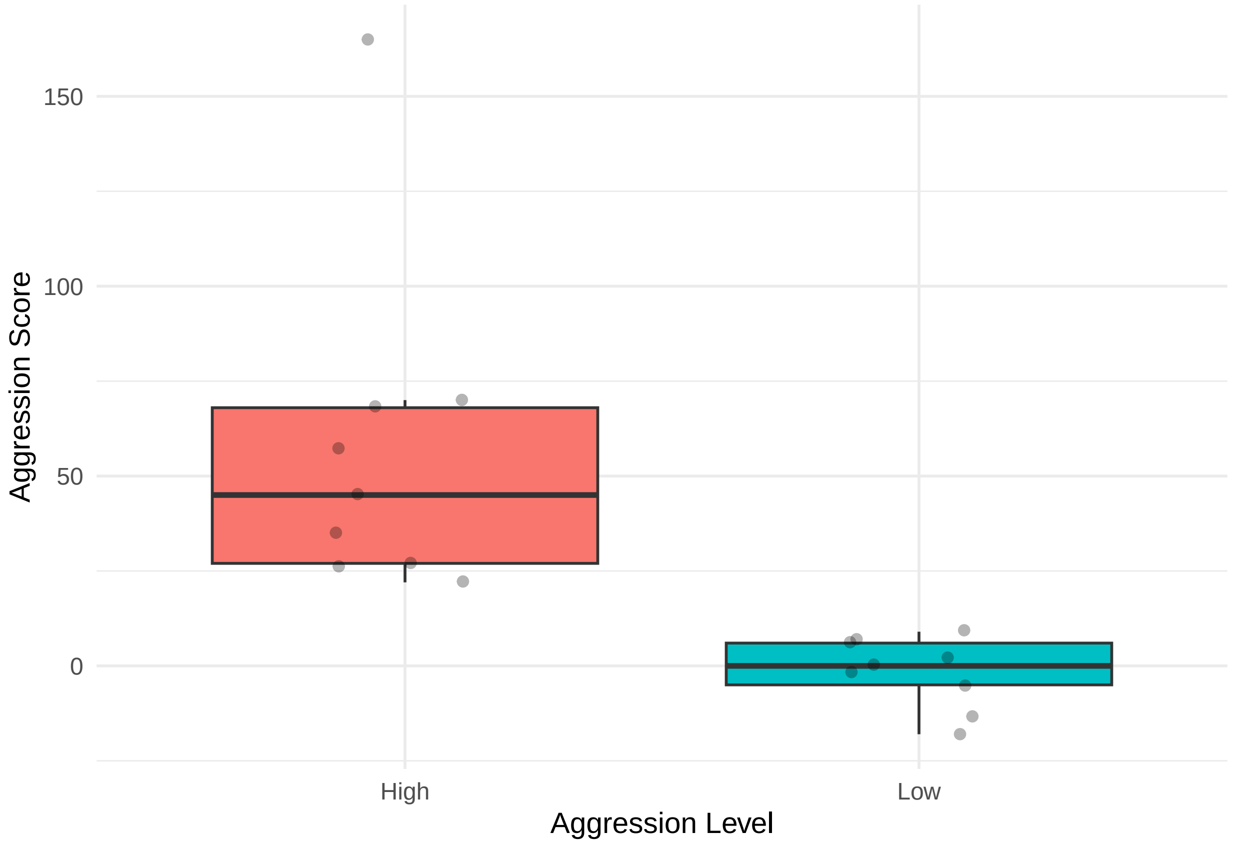

Figure S4, similar to Figure 3 but includes both total and relative (here called "Mass-Corrected") macronutrient values (again, so sorry for my very long, very messy code. . .)

```
plot_grid(ggdraw() + draw_plot(ggplot(WorkerJelly2025_Data, aes(x = Aggression,
    y = TotalDryWeight, fill = Aggression)) + geom_boxplot() +
    geom_point(position = position_jitterdodge(jitter.width = 0.4),
        alpha = 0.15) + xlab("Aggression") + ylab("Total Dry Weight (mg)") +
    theme_bw() + theme(strip.text = element_text(size = 12, face = "bold"),
    axis.text = element_text(size = 10), axis.title = element_text(size = 12,
        face = "bold")) + theme(legend.position = "none", axis.text.x = element_blank(),
    axis.title.x = element_blank()), x = 0, y = 0.5, width = 0.5,
    height = 0.5) + draw_plot(ggplot(WorkerJelly2025_Data, aes(x = Aggression,
    y = TotalProtein, fill = Aggression)) + geom_boxplot() +
    geom_point(position = position_jitterdodge(jitter.width = 0.4),
        alpha = 0.15) + xlab("Aggression") + ylab("Total Proteins (mg)") +
    theme_bw() + theme(strip.text = element_text(size = 12, face = "bold"),
    axis.text = element_text(size = 10), axis.title = element_text(size = 12,
        face = "bold")) + theme(legend.position = "none", axis.text.x = element_blank(),
    axis.title.x = element_blank()), x = 0.5, y = 0.5, width = 0.25,
```

```
    height = 0.5) + draw_plot(ggplot(WorkerJelly2025_Data, aes(x = Aggression,
    y = MassCorrectedProtein, fill = Aggression)) + geom_boxplot() +
    geom_point(position = position_jitterdodge(jitter.width = 0.4),
        alpha = 0.15) + xlab("Aggression") + ylab("Mass-Corrected Proteins (mg)") +
    theme_bw() + theme(strip.text = element_text(size = 12, face = "bold"),
    axis.text = element_text(size = 10), axis.title = element_text(size = 12,
        face = "bold")) + theme(legend.position = "none", axis.text.x = element_blank(),
    axis.title.x = element_blank()), x = 0.75, y = 0.5, width = 0.25,
    height = 0.5) + draw_plot(ggplot(WorkerJelly2025_Data, aes(x = Aggression,
    y = TotalLipids, fill = Aggression)) + geom_boxplot() + geom_point(position = position_jitterdodge(
    alpha = 0.15) + xlab("Aggression") + ylab("Total Lipids (mg)") +
    theme_bw() + theme(strip.text = element_text(size = 12, face = "bold"),
    axis.text = element_text(size = 10), axis.title = element_text(size = 12,
        face = "bold")) + theme(legend.position = "none", axis.text.x = element_blank(),
    axis.title.x = element_blank()), x = 0, y = 0, width = 0.25,
    height = 0.5) + draw_plot(ggplot(WorkerJelly2025_Data, aes(x = Aggression,
    y = MassCorrectedLipids, fill = Aggression)) + geom_boxplot() +
    geom_point(position = position_jitterdodge(jitter.width = 0.4),
        alpha = 0.15) + xlab("Aggression") + ylab("Mass-Corrected Lipids (mg)") +
    theme_bw() + theme(strip.text = element_text(size = 12, face = "bold"),
    axis.text = element_text(size = 10), axis.title = element_text(size = 12,
        face = "bold")) + theme(legend.position = "none", axis.text.x = element_blank(),
    axis.title.x = element_blank()), x = 0.25, y = 0, width = 0.25,
    height = 0.5) + draw_plot(ggplot(WorkerJelly2025_Data, aes(x = Aggression,
    y = TotalCarbs, fill = Aggression)) + geom_boxplot() + geom_point(position = position_jitterdodge(j
    alpha = 0.15) + xlab("Aggression") + ylab("Total Carbohydrates (mg)") +
    theme_bw() + theme(strip.text = element_text(size = 12, face = "bold"),
    axis.text = element_text(size = 10), axis.title = element_text(size = 12,
        face = "bold")) + theme(legend.position = "none", axis.text.x = element_blank(),
    axis.title.x = element_blank()), x = 0.5, y = 0, width = 0.25,
    height = 0.5) + draw_plot(ggplot(WorkerJelly2025_Data, aes(x = Aggression,
    y = MassCorrectedCarbs, fill = Aggression)) + geom_boxplot() +
    geom_point(position = position_jitterdodge(jitter.width = 0.4),
        alpha = 0.15) + xlab("Aggression") + ylab("Mass-Corrected Carbohydrates (mg)") +
    theme_bw() + theme(strip.text = element_text(size = 12, face = "bold"),
    axis.text = element_text(size = 10), axis.title = element_text(size = 12,
        face = "bold")) + theme(legend.position = "none", axis.text.x = element_blank(),
    axis.title.x = element_blank()), x = 0.75, y = 0, width = 0.25,
    height = 0.5) + draw_plot_label(label = c("A", "B", "C",
    "D", "E", "F", "G"), size = 15, x = c(0, 0.5, 0.75, 0, 0.25,
    0.5, 0.75), y = c(1, 1, 1, 0.5, 0.5, 0.5, 0.5)), get_legend((ggplot(WorkerJelly2025_Data,
    aes(x = Aggression, y = TotalDryWeight, fill = Aggression)) +
    geom_boxplot()) + guides(color = guide_legend(nrow = 1)) +
    theme(legend.position = "bottom")), ncol = 1, rel_heights = c(1,
    0.1))
```

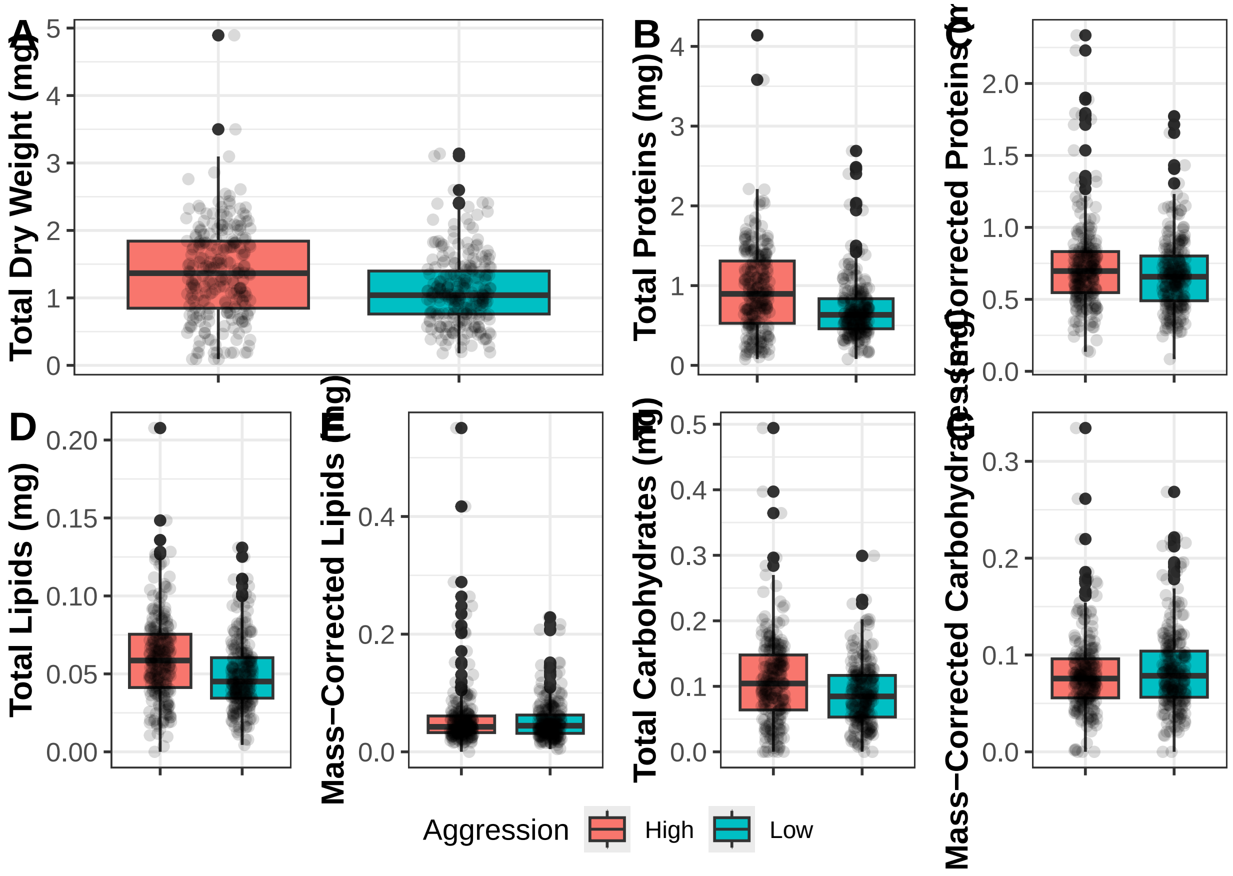

Figure S5, scatterplots of total dry weight and relative macronutrient quantities as a function of colony aggression rank

```
ggarrange(ggplot(WorkerJelly2025_Data, aes(x = AggRank_Centered,
    y = TotalDryWeight)) + geom_point() + geom_smooth(method = lm,
    color = "black") + theme_minimal() + xlab("Aggression Rank (centered on zero)") +
    ylab("Total Dry Mass (mg)") + theme(strip.text = element_text(size = 12,
    face = "bold"), axis.text = element_text(size = 10), axis.title = element_text(size = 12,
    face = "bold")) + theme(legend.position = "none", axis.title.x = element_blank()),
    ggplot(WorkerJelly2025_Data, aes(x = AggRank_Centered, y = MassCorrectedProtein)) +
        geom_point() + geom_smooth(method = lm, color = "black") +
        theme_minimal() + xlab("Aggression Rank (centered on zero)") +
        ylab("Relative Proteins") + theme(strip.text = element_text(size = 12,
        face = "bold"), axis.text = element_text(size = 10),
        axis.title = element_text(size = 12, face = "bold")) +
        theme(legend.position = "none", axis.title.x = element_blank()),
    ggplot(WorkerJelly2025_Data, aes(x = AggRank_Centered, y = MassCorrectedLipids)) +
        geom_point() + geom_smooth(method = lm, color = "black") +
        theme_minimal() + xlab("Aggression Rank (centered on zero)") +
        ylab("Relative Lipids") + theme(strip.text = element_text(size = 12,
        face = "bold"), axis.text = element_text(size = 10),
        axis.title = element_text(size = 12, face = "bold")) +
        theme(legend.position = "none"), ggplot(WorkerJelly2025_Data,
        aes(x = AggRank_Centered, y = MassCorrectedCarbs)) +
```

```
        geom_point() + geom_smooth(method = lm, color = "black") +
        theme_minimal() + xlab("Aggression Rank (centered on zero)") +
        ylab("Relative Carbohydrates") + theme(strip.text = element_text(size = 12,
        face = "bold"), axis.text = element_text(size = 10),
        axis.title = element_text(size = 12, face = "bold")) +
        theme(legend.position = "none"), labels = c("A", "B",
        "C", "D"), ncol = 2, nrow = 2)
```

```
## `geom_smooth()` using formula = 'y ~ x'
## `geom_smooth()` using formula = 'y ~ x'
## `geom_smooth()` using formula = 'y ~ x'
## `geom_smooth()` using formula = 'y ~ x'
```

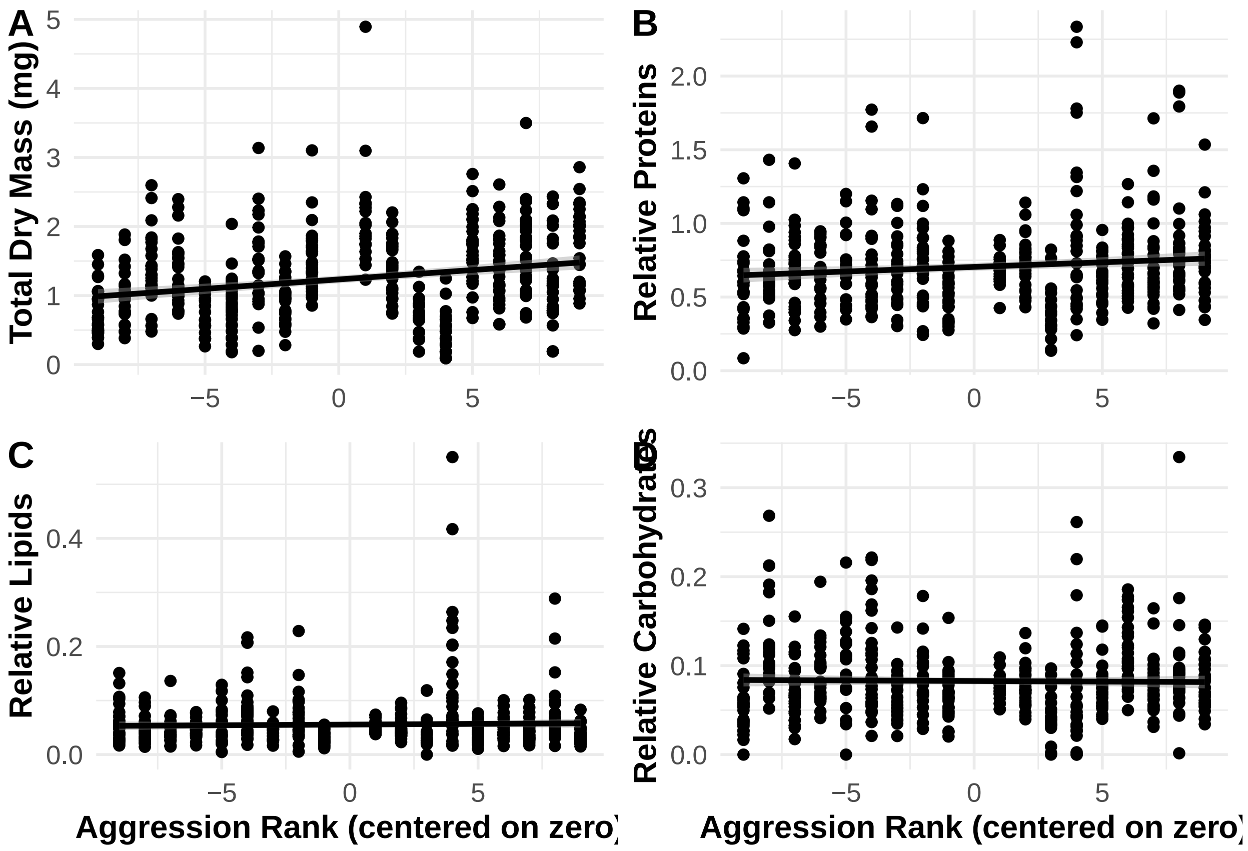

Figure S6, scatterplot of total dry masses and macronutrient masses as a function of aggression rank, split by aggression level

```
ggarrange(ggplot(WorkerJelly2025_Data, aes(x = AggRank_Centered,
    y = TotalDryWeight, fill = Aggression)) + geom_point(aes(color = Aggression)) +
    geom_smooth(method = lm, color = "black") + theme_minimal() +
    xlab("Aggression Rank (centered on zero)") + ylab("Total Dry Mass (mg)") +
    theme(strip.text = element_text(size = 12, face = "bold"),
        axis.text = element_text(size = 10), axis.title = element_text(size = 12,
```

```
        face = "bold")) + theme(legend.position = "none",
    axis.title.x = element_blank()), ggplot(WorkerJelly2025_Data,
    aes(x = AggRank_Centered, y = TotalProtein, fill = Aggression)) +
    geom_point(aes(color = Aggression)) + geom_smooth(method = lm,
    color = "black") + theme_minimal() + xlab("Aggression Rank (centered on zero)") +
    ylab("Total Protein Mass (mg)") + theme(strip.text = element_text(size = 12,
    face = "bold"), axis.text = element_text(size = 10), axis.title = element_text(size = 12,
    face = "bold")) + theme(legend.position = "none", axis.title.x = element_blank()),
    ggplot(WorkerJelly2025_Data, aes(x = AggRank_Centered, y = TotalLipids,
        fill = Aggression)) + geom_point(aes(color = Aggression)) +
        geom_smooth(method = lm, color = "black") + theme_minimal() +
        xlab("Aggression Rank (centered on zero)") + ylab("Total Lipid Mass (mg)") +
        theme(strip.text = element_text(size = 12, face = "bold"),
            axis.text = element_text(size = 10), axis.title = element_text(size = 12,
                face = "bold")) + theme(legend.position = "none"),
    ggplot(WorkerJelly2025_Data, aes(x = AggRank_Centered, y = TotalCarbs,
        fill = Aggression)) + geom_point(aes(color = Aggression)) +
        geom_smooth(method = lm, color = "black") + theme_minimal() +
        xlab("Aggression Rank (centered on zero)") + ylab("Total Carbohydrate Mass (mg)") +
        theme(strip.text = element_text(size = 12, face = "bold"),
            axis.text = element_text(size = 10), axis.title = element_text(size = 12,
                face = "bold")) + theme(legend.position = "none"),
    labels = c("A", "B", "C", "D"), ncol = 2, nrow = 2, common.legend = TRUE,
    legend = "bottom")
```

```
## `geom_smooth()` using formula = 'y ~ x'
## `geom_smooth()` using formula = 'y ~ x'
## `geom_smooth()` using formula = 'y ~ x'
## `geom_smooth()` using formula = 'y ~ x'
## `geom_smooth()` using formula = 'y ~ x'
```

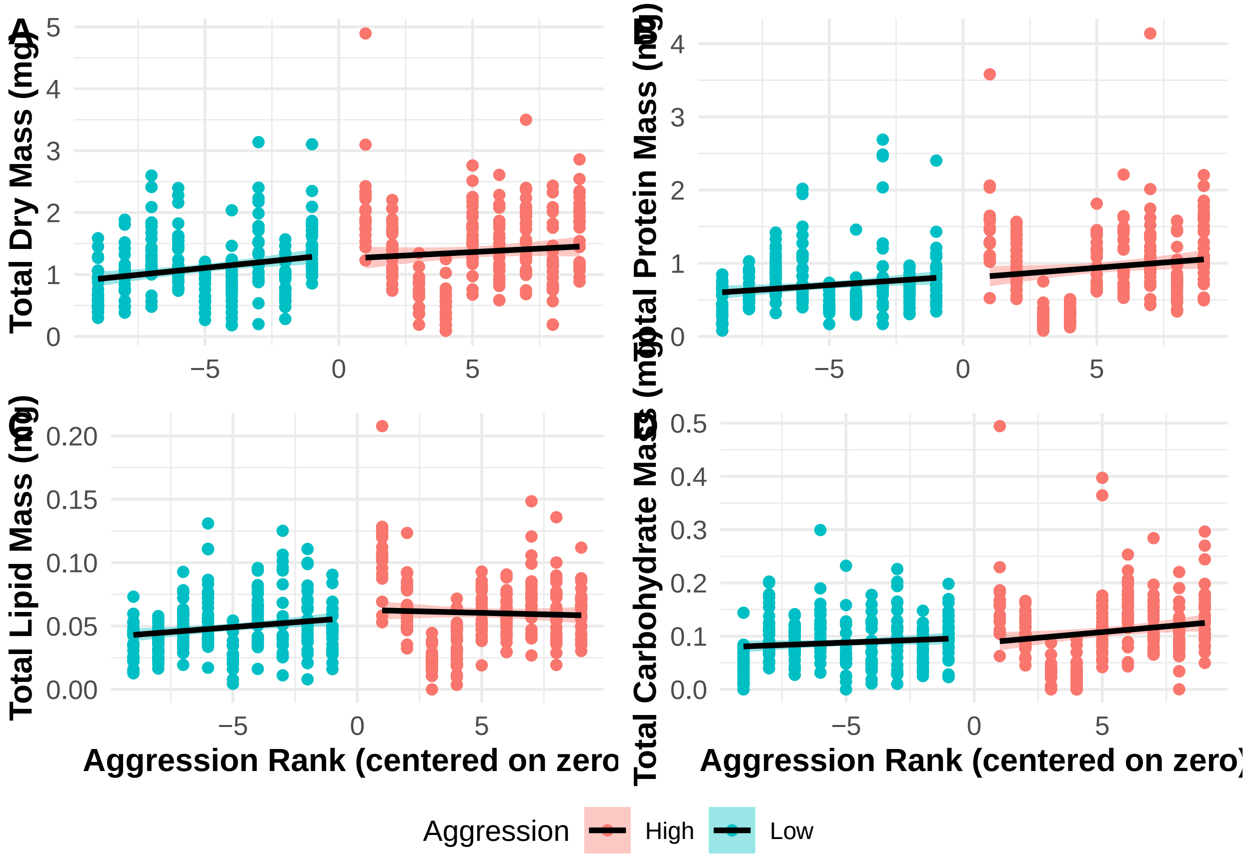

Figure S7, scatterplot of total dry masses and macronutrient masses as a function of raw aggression score, split by aggression level

```
ggarrange(ggplot(WorkerJelly2025_Data, aes(x = AggScore, y = TotalDryWeight,
    fill = Aggression)) + geom_point(aes(color = Aggression)) +
    geom_smooth(method = lm, color = "black") + theme_minimal() +
    xlab("Aggression Rank (centered on zero)") + ylab("Total Dry Mass (mg)") +
    theme(strip.text = element_text(size = 12, face = "bold"),
        axis.text = element_text(size = 10), axis.title = element_text(size = 12,
            face = "bold")) + theme(legend.position = "none",
    axis.title.x = element_blank()), ggplot(WorkerJelly2025_Data,
    aes(x = AggScore, y = TotalProtein, fill = Aggression)) +
    geom_point(aes(color = Aggression)) + geom_smooth(method = lm,
    color = "black") + theme_minimal() + xlab("Aggression Rank (centered on zero)") +
    ylab("Total Protein Mass (mg)") + theme(strip.text = element_text(size = 12,
    face = "bold"), axis.text = element_text(size = 10), axis.title = element_text(size = 12,
    face = "bold")) + theme(legend.position = "none", axis.title.x = element_blank()),
    ggplot(WorkerJelly2025_Data, aes(x = AggScore, y = TotalLipids,
        fill = Aggression)) + geom_point(aes(color = Aggression)) +
        geom_smooth(method = lm, color = "black") + theme_minimal() +
        xlab("Raw Aggression Score") + ylab("Total Lipid Mass (mg)") +
        theme(strip.text = element_text(size = 12, face = "bold"),
            axis.text = element_text(size = 10), axis.title = element_text(size = 12,
                face = "bold")) + theme(legend.position = "none"),
```

```
    ggplot(WorkerJelly2025_Data, aes(x = AggScore, y = TotalCarbs,
        fill = Aggression)) + geom_point(aes(color = Aggression)) +
        geom_smooth(method = lm, color = "black") + theme_minimal() +
        xlab("Raw Aggression Score") + ylab("Total Carbohydrate Mass (mg)") +
        theme(strip.text = element_text(size = 12, face = "bold"),
            axis.text = element_text(size = 10), axis.title = element_text(size = 12,
                face = "bold")) + theme(legend.position = "none"),
    labels = c("A", "B", "C", "D"), ncol = 2, nrow = 2, common.legend = TRUE,
    legend = "bottom")
```

```
## `geom_smooth()` using formula = 'y ~ x'
## `geom_smooth()` using formula = 'y ~ x'
## `geom_smooth()` using formula = 'y ~ x'
## `geom_smooth()` using formula = 'y ~ x'
## `geom_smooth()` using formula = 'y ~ x'
```

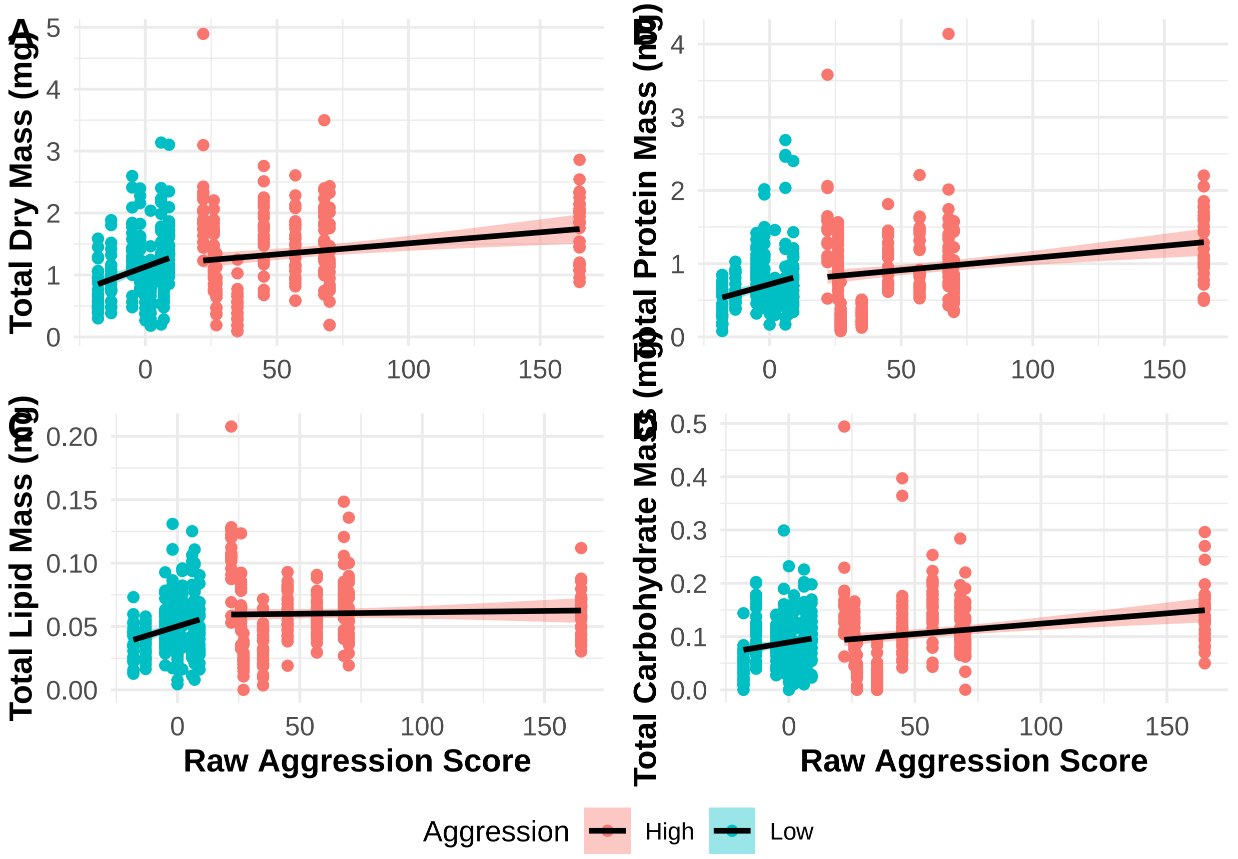

Figure S8, line plot of total dry mass and relative macronutrient masses versus experiment day

```
ggarrange(ggplot(ColonyAggression, aes(x = Day, y = TotalDryWeight,
    group = Aggression)) + geom_line(aes(linetype = Aggression)) +
    geom_point(aes(shape = Aggression)) + theme_minimal() + ylab("Total Dry Mass (mg)"),
```

```
    ggplot(ColonyAggression, aes(x = Day, y = MassCorrectedProtein,
        group = Aggression)) + geom_line(aes(linetype = Aggression)) +
        geom_point(aes(shape = Aggression)) + theme_minimal() +
        ylab("Relative Proteins"), ggplot(ColonyAggression, aes(x = Day,
        y = MassCorrectedLipids, group = Aggression)) + geom_line(aes(linetype = Aggression)) +
        geom_point(aes(shape = Aggression)) + theme_minimal() +
        ylab("Relative Lipids"), ggplot(ColonyAggression, aes(x = Day,
        y = MassCorrectedCarbs, group = Aggression)) + geom_line(aes(linetype = Aggression)) +
        geom_point(aes(shape = Aggression)) + theme_minimal() +
        ylab("Relative Carbohydrates"), labels = c("A", "B",
        "C", "D"), ncol = 2, nrow = 2)
```

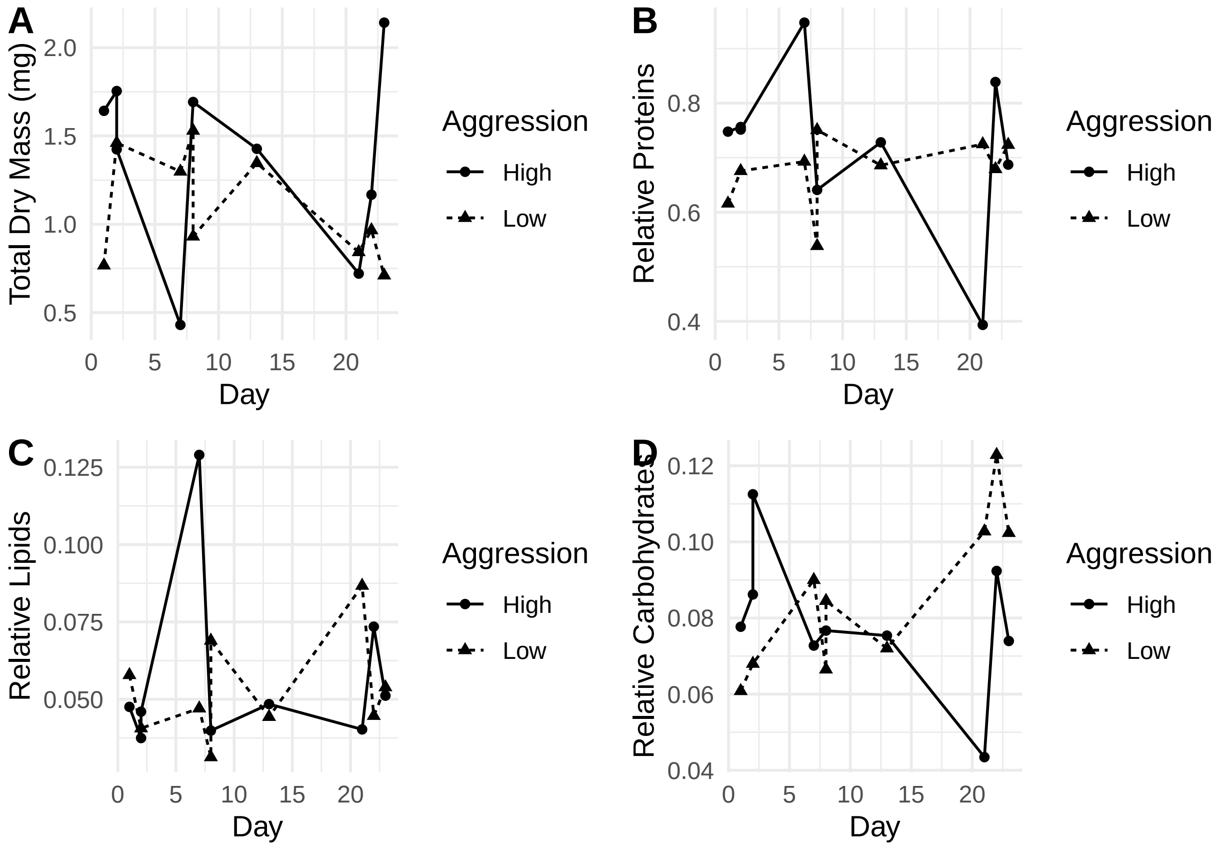

Figure S9, boxplots of total dry mass and relative macronutrient quantities by colony genetic strain

Creating boxplots

```
SiteBoxplots_Relative <- ggdraw() + draw_plot(ggplot(WorkerJelly2025_Data,
    aes(x = factor(Location, level = c("Alpha", "Beta", "Gamma")),
        y = TotalDryWeight, fill = Location)) + geom_boxplot() +
    geom_point(position = position_jitterdodge(jitter.width = 0.9),
        alpha = 0.15) + xlab("Site") + ylab("Total Dry Mass (mg)") +
    scale_fill_viridis(discrete = TRUE, begin = 0.25) + theme_bw() +
```

```
    theme(axis.text = element_text(size = 10), axis.title = element_text(size = 12,
        face = "bold")) + theme(legend.position = "none", axis.title.x = element_blank(),
    axis.text.x = element_blank()), x = 0, y = 0.5, width = 0.5,
    height = 0.5) + draw_plot(ggplot(WorkerJelly2025_Data, aes(x = factor(Location,
    level = c("Alpha", "Beta", "Gamma")), y = MassCorrectedProtein,
    fill = Location)) + geom_boxplot() + geom_point(position = position_jitterdodge(jitter.width = 0.9)
    alpha = 0.15) + xlab("Site") + ylab("Relative Proteins") +
    scale_fill_viridis(discrete = TRUE, begin = 0.25) + theme_bw() +
    theme(axis.text = element_text(size = 10), axis.title = element_text(size = 12,
        face = "bold")) + theme(legend.position = "none", axis.title.x = element_blank(),
    axis.text.x = element_blank()), x = 0.5, y = 0.5, width = 0.5,
    height = 0.5) + draw_plot(ggplot(WorkerJelly2025_Data, aes(x = factor(Location,
    level = c("Alpha", "Beta", "Gamma")), y = MassCorrectedLipids,
    fill = Location)) + geom_boxplot() + geom_point(position = position_jitterdodge(jitter.width = 0.9)
    alpha = 0.15) + xlab("Site") + ylab("Relative Lipids") +
    scale_fill_viridis(discrete = TRUE, begin = 0.25) + theme_bw() +
    theme(axis.text = element_text(size = 10), axis.title = element_text(size = 12,
        face = "bold")) + theme(legend.position = "none", axis.title.x = element_blank(),
    axis.text.x = element_blank()), x = 0, y = 0, width = 0.5,
    height = 0.5) + draw_plot(ggplot(WorkerJelly2025_Data, aes(x = factor(Location,
    level = c("Alpha", "Beta", "Gamma")), y = MassCorrectedCarbs,
    fill = Location)) + geom_boxplot() + geom_point(position = position_jitterdodge(jitter.width = 0.9)
    alpha = 0.15) + xlab("Site") + ylab("Relative Carbohydrates") +
    scale_fill_viridis(discrete = TRUE, begin = 0.25) + theme_bw() +
    theme(axis.text = element_text(size = 10), axis.title = element_text(size = 12,
        face = "bold")) + theme(legend.position = "none", axis.title.x = element_blank(),
    axis.text.x = element_blank()), x = 0.5, y = 0, width = 0.5,
    height = 0.5) + draw_plot_label(label = c("A", "B", "C",
    "D"), size = 15, x = c(0, 0.5, 0, 0.5), y = c(1, 1, 0.5,
    0.5))
```

Adding legend

```
plot_grid(SiteBoxplots_Relative, get_legend((ggplot(WorkerJelly2025_Data,
    aes(x = factor(Location, level = c("Alpha", "Beta", "Gamma")),
        y = TotalDryWeight, fill = Location)) + geom_boxplot() +
    scale_fill_viridis(discrete = TRUE, begin = 0.25, name = "Site",
        breaks = c("Alpha", "Beta", "Gamma"))) + guides(color = guide_legend(nrow = 1)) +
    theme(legend.position = "bottom")), ncol = 1, rel_heights = c(1,
    0.1))
```

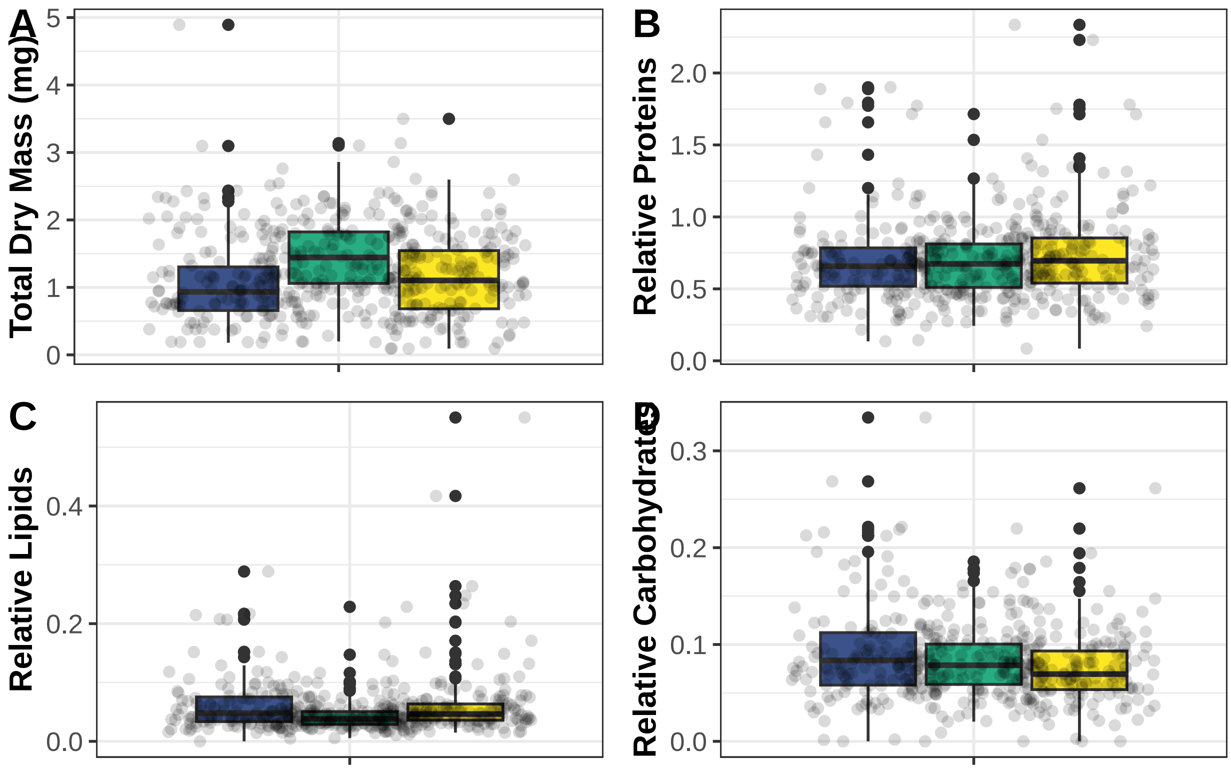

Figure S10, boxplots of total dry mass and relative macronutrient quantities by site

Creating boxplots

```
StrainBoxplots_Relative <- ggdraw() + draw_plot(ggplot(WorkerJelly2025_Data_Source,
    aes(x = Source, y = TotalDryWeight, fill = Source)) + geom_boxplot() +
    geom_point(position = position_jitterdodge(jitter.width = 0.4),
        alpha = 0.15) + xlab("Strain") + ylab("Total Dry Mass (mg)") +
    scale_fill_viridis(discrete = TRUE, begin = 0.35, end = 0.9,
        option = "magma") + theme_bw() + theme(axis.text = element_text(size = 10),
    axis.title = element_text(size = 12, face = "bold")) + theme(legend.position = "none",
    axis.title.x = element_blank(), axis.text.x = element_blank()),
    x = 0, y = 0.5, width = 0.5, height = 0.5) + draw_plot(ggplot(WorkerJelly2025_Data_Source,
    aes(x = Source, y = MassCorrectedProtein, fill = Source)) +
    geom_boxplot() + geom_point(position = position_jitterdodge(jitter.width = 0.4),
    alpha = 0.15) + xlab("Strain") + ylab("Relative Proteins") +
    scale_fill_viridis(discrete = TRUE, begin = 0.35, end = 0.9,
        option = "magma") + theme_bw() + theme(axis.text = element_text(size = 10),
    axis.title = element_text(size = 12, face = "bold")) + theme(legend.position = "none",
    axis.title.x = element_blank(), axis.text.x = element_blank()),
    x = 0.5, y = 0.5, width = 0.5, height = 0.5) + draw_plot(ggplot(WorkerJelly2025_Data_Source,
    aes(x = Source, y = MassCorrectedLipids, fill = Source)) +
    geom_boxplot() + geom_point(position = position_jitterdodge(jitter.width = 0.4),
```

```
    alpha = 0.15) + xlab("Strain") + ylab("Relative Lipids") +
    scale_fill_viridis(discrete = TRUE, begin = 0.35, end = 0.9,
        option = "magma") + theme_bw() + theme(axis.text = element_text(size = 10),
    axis.title = element_text(size = 12, face = "bold")) + theme(legend.position = "none",
    axis.title.x = element_blank(), axis.text.x = element_blank()),
    x = 0, y = 0, width = 0.5, height = 0.5) + draw_plot(ggplot(WorkerJelly2025_Data_Source,
    aes(x = Source, y = MassCorrectedCarbs, fill = Source)) +
    geom_boxplot() + geom_point(position = position_jitterdodge(jitter.width = 0.4),
    alpha = 0.15) + xlab("Strain") + ylab("Relative Carbohydrates") +
    scale_fill_viridis(discrete = TRUE, begin = 0.35, end = 0.9,
        option = "magma") + theme_bw() + theme(axis.text = element_text(size = 10),
    axis.title = element_text(size = 12, face = "bold")) + theme(legend.position = "none",
    axis.title.x = element_blank(), axis.text.x = element_blank()),
    x = 0.5, y = 0, width = 0.5, height = 0.5) + draw_plot_label(label = c("A",
    "B", "C", "D"), size = 15, x = c(0, 0.5, 0, 0.5), y = c(1,
    1, 0.5, 0.5))
```

Adding legend

```
plot_grid(StrainBoxplots_Relative, get_legend((ggplot(WorkerJelly2025_Data_Source,
    aes(x = factor(Source), y = TotalDryWeight, fill = Source)) +
    geom_boxplot() + scale_fill_viridis(discrete = TRUE, begin = 0.35,
    end = 0.9, option = "magma", name = "Strain")) + guides(color = guide_legend(nrow = 1)) +
    theme(legend.position = "bottom")), ncol = 1, rel_heights = c(1,
    0.1))
```

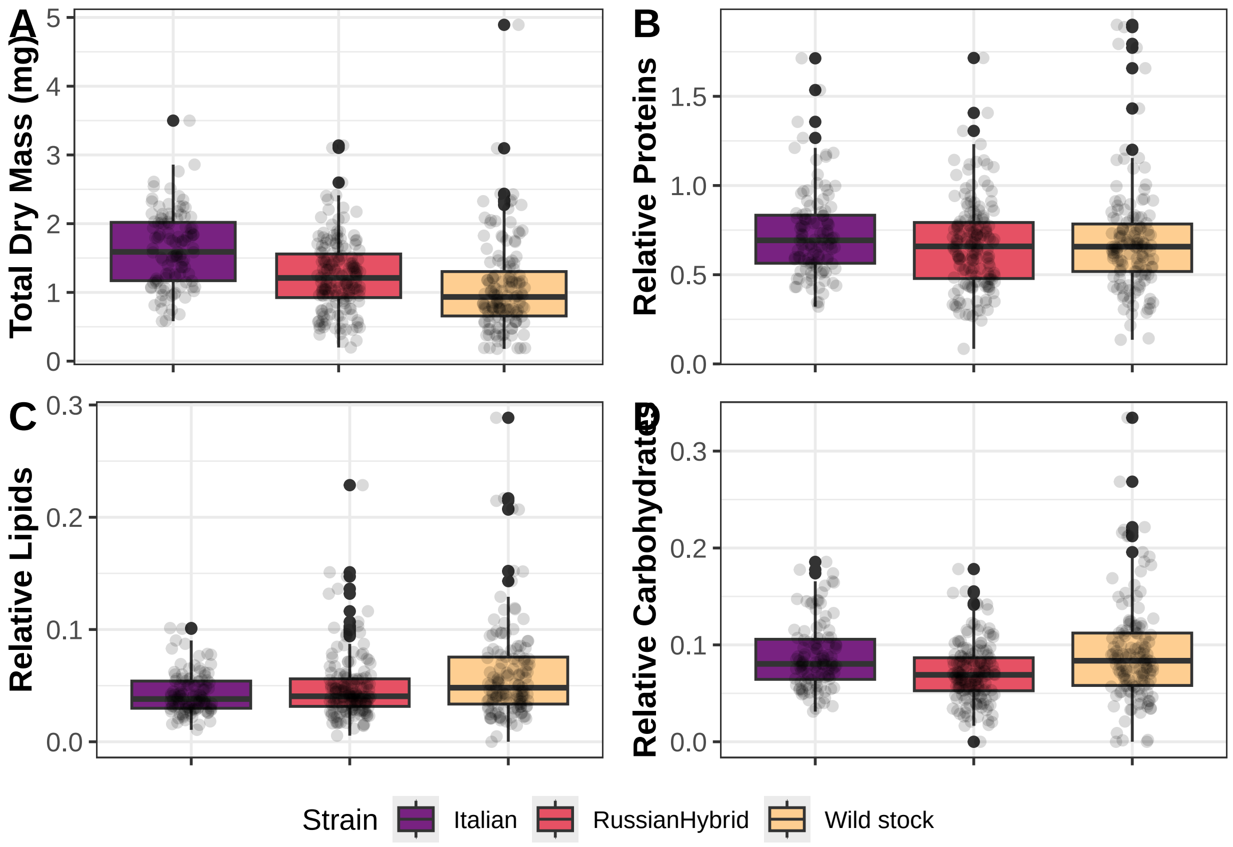

Thank you for reading! Feel free to reach out if you have any questions!
